# Human umbilical cord-derived mesenchymal stem cell transplantation supplemented with curcumin improves the outcomes of ischemic stroke via AKT/GSK-3β/β-TrCP/Nrf2 axis

**DOI:** 10.1186/s12974-023-02738-5

**Published:** 2023-02-24

**Authors:** Yuan Li, Jialu Huang, Jie Wang, Simin Xia, Hong Ran, Lenyu Gao, Chengjian Feng, Li Gui, Zhenhua Zhou, Jichao Yuan

**Affiliations:** 1 Department of Neurology, Southwest Hospital, Third Military Medical University (Army Medical University), 29 Gaotanyan Street, Chongqing, 400038 China; 2 Department of Traditional Chinese Medicine and Rheumatology, Southwest Hospital, Third Military Medical University (Army Medical University), Chongqing, 400038 China; 3 Department of Medical Engineering, 958th Hospital of the People’s Liberation Army, Chongqing, 400038 China

**Keywords:** Ischemic stroke, Human umbilical cord-derived mesenchymal stem cells, Curcumin, Anti-inflammatory phenotype microglia, AKT/GSK-3β/β-TrCP/Nrf2 axis

## Abstract

**Background:**

Human umbilical cord-derived mesenchymal stem cell (hUC-MSC) engraftment is a promising therapy for acute ischemic stroke (AIS). However, the harsh ischemic microenvironment limits the therapeutic efficacy of hUC-MSC therapy. Curcumin is an anti-inflammatory agent that could improve inflammatory microenvironment. However, whether it enhances the neuroprotective efficacy of hUC-MSC transplantation is still unknown. In the present study, we investigated the therapeutic efficacy and the possible mechanism of combined curcumin and hUC-MSC treatment in AIS.

**Methods:**

Middle cerebral artery occlusion (MCAO) mice and oxygen glucose deprivation (OGD) microglia were administrated hUC-MSCs with or without curcumin. Neurological deficits assessment, brain water content and TTC were used to assess the therapeutic effects of combined treatment. To elucidate the mechanism, MCAO mice and OGD microglia were treated with AKT inhibitor MK2206, GSK3β activator sodium nitroprusside (SNP), GSK3β inhibitor TDZD-8 and *Nrf2 gene* knockout were used. Immunofluorescence, flow cytometric analysis, WB and RT-PCR were used to evaluate the microglia polarization and the expression of typical oxidative mediators, inflammatory cytokines and the AKT/GSK-3β/β-TrCP/Nrf2 pathway protein.

**Results:**

Compared with the solo hUC-MSC-grafted or curcumin groups, combined curcumin-hUC-MSC therapy significantly improved the functional performance outcomes, diminished the infarct volumes and the cerebral edema. The combined treatment promoted anti-inflammatory microglia polarization via Nrf2 pathway and decreased the expression of ROS, oxidative mediators and pro-inflammatory cytokines, while elevating the expression of the anti-inflammatory cytokines. *Nrf2* knockout abolished the antioxidant stress and anti-inflammation effects mediated with combined treatment. Moreover, the combined treatment enhanced the phosphorylation of AKT and GSK3β, inhibited the β-TrCP nucleus translocation, accompanied with Nrf2 activation in the nucleus. AKT inhibitor MK2206 activated GSK3β and β-TrCP and suppressed Nrf2 phosphorylation in nucleus, whereas MK2206 with the GSK3β inhibitor TDZD-8 reversed these phenomena. Furthermore, combined treatment followed by GSK3β inhibition with TDZD-8 restricted β-TrCP nucleus accumulation, which facilitated Nrf2 expression.

**Conclusions:**

We have demonstrated that combined curcumin-hUC-MSC therapy exerts anti-inflammation and antioxidant stress efficacy mediated by anti-inflammatory microglia polarization via AKT/GSK-3β/β-TrCP/Nrf2 axis and an improved neurological function after AIS.

**Supplementary Information:**

The online version contains supplementary material available at 10.1186/s12974-023-02738-5.

## Introduction

Acute ischemic stroke (AIS) is a leading cause of death and severe disability worldwide [1]. Loss of function after stroke is due to inflammation-mediated neuronal death in ischemic penumbra accompanied by primary injury. Suppressing inflammation is an effective strategy for stroke treatment [2]. Currently, there is an increased focus on stem cell therapy research, especially mesenchymal stem cells (MSCs) in treating AIS. Previous studies have revealed that MSCs possess powerful differentiation potential in vitro indicating that the grafted MSCs would differentiate into mature functional neural lineage cells, integrate into the host network and repair damaged tissues [3, 4]. However, now transplanted MSCs are believed to provide an appropriate anti-inflammatory microenvironment for tissue repair rather than cell replacement[5]. Additionally, MSCs promote AIS recovery through angiogenesis, secretion of neurotrophic factors, inhibition of apoptosis, and modulation of the immune system[6]. These advantages make MSCs a prime candidate for AIS therapy.

Compared with other types of MSCs, human umbilical cord-derived mesenchymal stem cells (hUC-MSCs) have important advantages. Firstly, their use does not violate ethical concerns. In addition, hUC-MSCs have low oncogenicity, exhibit easy in vitro expansion, and have higher immunomodulatory capacity [7, 8]. Therefore, hUC-MSCs serve as the preferred seed cells for stem cell therapy. There have been many animal studies as well as preclinical trials exploring the therapeutic efficacy of hUC-MSCs in AIS [9–12]. However, transplantation of hUC-MSCs into the ischemic area has not always yielded satisfactory locomotor outcomes because of low engraftment and survival rates of hUC-MSCs resulting from harsh inflammatory microenvironment. Thus, their clinical application is restricted [13]. Consequently, it is imperative to develop appropriate methods for improving the therapeutic efficacy of engrafted hUC-MSCs.

Curcumin, an active ingredient of the perennial turmeric herb, possesses neuroprotective activity in the nervous system. Our laboratory has confirmed that curcumin exerts anti-inflammatory effects in spinal cord injury [14, 15], traumatic brain injury [16] and intracerebral hemorrhage [17, 18]. Several studies have also reported that curcumin ameliorates neurological impairment through its anti-inflammatory, antioxidant and anti-apoptotic properties, and alleviates blood–brain barrier integrity [19–21]. Nevertheless, it remains unknown whether curcumin could enhance the therapeutic efficacy of engrafted hUC-MSCs in AIS.

Nuclear factor erythroid 2-related factor 2 (Nrf2) is a “master regulator” of the antioxidant response and has been shown to play a critical role in microglia/macrophages phenotype polarization [22]. In physiological circumstances, Nrf2 binds to its negative regulator Kelch-like ECH-associated protein 1 (Keap1) which leads to Nrf2 degradation via ubiquitination. In response to stimuli such as inflammation and injury, Nrf2 is dissociated from Keap1 and translocated to the nucleus, where it binds to genes containing antioxidant response elements (ARE) to execute the anti-inflammatory and antioxidant function [23]. Besides Keap1, glycogen synthase kinase 3 beta (GSK3β) is another regulator of Nrf2 that acts via a Keap1-independent pathway, and activates β-transducin repeat-containing protein (β-TrCP) by ubiquitination and translocates it into the nucleus to degrade Nrf2 by phosphorylation of serine residues in the Neh6 domain [24]. However, whether hUC-MSCs transplantation along with curcumin treatment can suppress inflammation through the GSK3β/β-TrCP/Nrf2 pathway in AIS is still unclear.

**Fig. 1:**
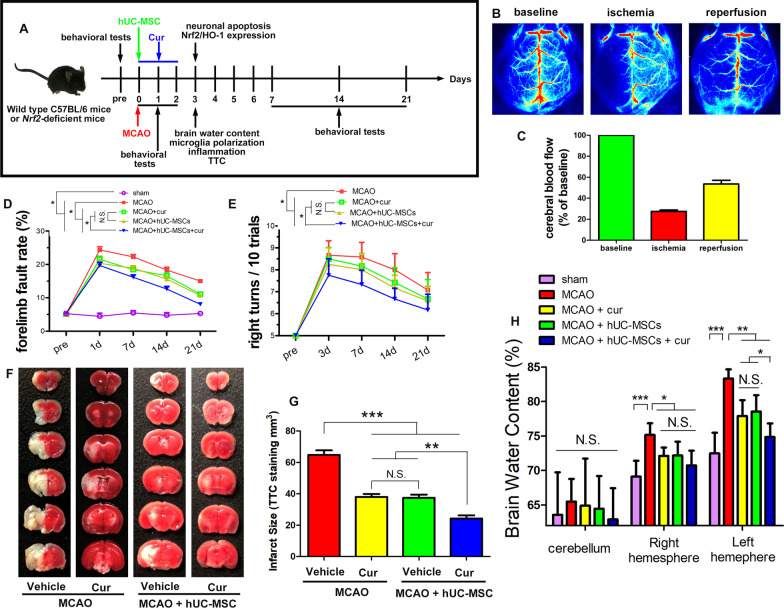
Combined curcumin-hUC-MSC treatment promoted functional outcomes in MCAO mice. **A** The scheme of the experimental timeline of this research. **B**, **C** Representative images (**B**) and the quantification (**C**) of cerebral blood flow before cerebral ischemia, 5 min after ischemia, and 5 min after reperfusion with laser Doppler flowmetry. **D**, **E** The grid-walking test and the corner test were performed to assess neurological deficits. There were no significant differences in the forelimb fault rate (**D**) and right turns rate (**E**) between the MCAO + hUC-MSCs and MCAO + curcumin groups at each time point. MCAO + hUC-MSCs + curcumin group significantly improved the neurological deficit compared with other groups. N = 12. **F**, **G** TTC-stained for brain sections (**F**) and quantitative data (**G**) showed the decreased infarct volume after curcumin combined hUC-MSC transplantation, N = 8. **H** The brain water content of the cerebellum, ipsilateral, contralateral of infarct hemisphere, N = 8. *Cur* curcumin, *N.S.* no significant difference, *P < 0.05, **P < 0.01, ***P < 0.001

We hypothesized that combined curcumin treatment might enhance hUC-MSC therapeutic efficacy in AIS. To verify this hypothesis, we studied the effect of peritoneal curcumin injection on transplantation efficacy of intravenously delivered hUC-MSC in an AIS murine model with transient middle cerebral artery occlusion (MCAO). We assessed motor function recovery in MCAO mice undergoing a combined hUC-MSC and curcumin therapy. We further investigated the possible mechanisms involved in the effects of the combined curcumin-hUC-MSC therapy in MCAO mice (Fig. 1A).

## Materials and methods

### Administration of the reagents and the hUC-MSCs

Curcumin (St. Louis, MO, Lot: C1386) was administered via intraperitoneal injection, and 100 mg of curcumin was dissolved in 1 mL dimethyl sulfoxide (DMSO) and 0.5 mL saline [25]. The first injection was administered immediately after MCAO and then injections were performed once every 24 h for 3 d. To treat cells, a dose of 4 μmol/L curcumin was selected. The hUC-MSCs (passage number 2 to 3) were collected and suspended in 0.2 mL saline at 10^6^ cells/20 g mouse and intravenously injected into tail veins at 0.1 mL/min 30 min after MCAO. The doses of AKT inhibitor 8-[4-(1-aminocyclobutyl)phenyl]-9-phenyl-2*H*-[1,2,4]triazolo[3,4-f][1,6]naphthyridin-3-one (MK2206, Cat# HY-10358, MedChemExpress, Monmouth Junction, USA) were 5 mg/kg intraperitoneally for mice and 5 μM for cells, those of the GSK3β activator sodium nitroprusside (SNP, Cat# HY-B0564, MedChemExpress) were 5 mg/kg intraperitoneally for mice and 100 μM for cells, and for the GSK-3β inhibitor 4-Benzyl-2-methyl-1,2,4-thiadiazolidine-3,5-dione, (TDZD-8, Cat# HY-11012, MedChemExpress), they were 5 mg/kg intraperitoneally for mice and 5 μM for cells.

### Culture and identification of hUC-MSCs

The clinical-grade hUC-MSCs were supplied by Chongqing Guolian Stem Cell Technology Co., Ltd. The cells were suspended in mesenchymal stem cell basal medium (MSCBM) supplemented with 5% UltraGROTM-Advanced (AventaCell, HPCFDCRL05) [25]. Fluorescence-activated cell sorting (FACS) analysis for hUC-MSC-specific markers (CD73, CD90, and CD105) and hematopoietic stem cell-specific markers (CD34, CD45, and HLA-DR) were performed to identify the immunophenotype.

### Animals

Adult male C57BL/6 mice (6–8 weeks, 22–25 g) were provided by the Animal Center of Third Military Medical University. *Nrf2*-deficient ICR mice were donated by Prof. Handong Wang (Department of Neurosurgery, Jinling Hospital, School of Medicine, Nanjing University, People's Republic of China) with permission from Dr. Thomas W. Kensler (Johns Hopkins University, Baltimore, MD, USA). *Nrf2* (+/+) (Wild-type, WT) and *Nrf2* (−/−) (knockout, KO) ICR mice were bred from heterozygous of *Nrf2* ( ±) Institute of Cancer Research (ICR) mice. The mice had ad libitum food and water and were housed within a room with a 12:12 h dark–light cycle. All animal experiments were performed in accordance with China’s animal welfare legislation for the care and use of animals and approved by the Army Military Medical University (No. AMUWEC20171590). Middle cerebral artery occlusion was performed to induce AIS in the mice.

For the first part of the experiment, the C57BL/6 mice were divided into five groups as follows: (1) sham group, (2) MCAO group, (3) MCAO + curcumin, (4) MCAO + hUC-MSCs, and (5) MCAO + hUC-MSCs + curcumin group. Mice that did not receive MCAO operation were defined as the sham group and were injected with 0.04 mL of DMSO intraperitoneally and 0.2 mL of saline intravenously. The MCAO group mice were injected with 0.04 mL of DMSO intraperitoneally and 0.2 mL of saline intravenously after MCAO. The MCAO + curcumin group mice were intraperitoneally injected with 100 mg/kg curcumin (approximately 0.04 mL DMSO) and intravenously injected with 0.2 mL of saline [25]. The mice in the MCAO + hUC-MSCs group were transplanted with hUC-MSCs and 0.04 mL of DMSO intraperitoneally post MCAO. The mice in the MCAO + hUC-MSCs + curcumin group were intravenously injected with hUC-MSCs combined with 100 mg/kg curcumin intraperitoneally. For experiments conducted to elucidate the mechanism, the ICR mice were randomly divided into nine groups: (1) sham (WT), (2) MCAO (WT), (3) treatment (WT), (4) MCAO + treatment + MK2206 (WT), (5) MCAO + treatment + SNP, (6) MCAO + treatment + MK2206 + TDZD-8 (WT), (7) sham (KO), (8) MCAO (KO), and (9) treatment (KO). Mice in the treatment groups were given the combined treatment with the curcumin and hUC-MSCs. The MK2206, SNP and TDZD-8 were injected intraperitoneally for 3 consecutive days after MCAO and the first injection was administered immediately after MCAO. All the dosing procedures were consistent with curcumin.

### Transient middle cerebral artery occlusion (MCAO) in mice

To establish cerebral ischemia MCAO, mice were anesthetized by isoflurane (3% for induction and 1.5% for maintenance) inhalation [26]. The left common and external carotid arteries were isolated and ligated. A microvascular clip was temporarily placed on the internal carotid artery. A 6–0 nylon suture coated with a rounded tip was introduced through a small incision into the internal carotid arteries via the external carotid arteries and advanced 5 mm distal to the carotid bifurcation for MCAO. After 90 min of ischemia, the filament was withdrawn to allow reperfusion. Sham-operated mice were subjected to the same surgical procedure, except for filament insertion. The rectal temperature was maintained between 36 °C and 37 °C using a feedback-controlled heating system. Regional cerebral blood flow (CBF) was monitored using laser Doppler flowmetry (Fig. 1B, C). Mice were excluded from further experiment if the CBF failed to decrease to < 30% of pre-ischemia baseline levels.

### Neurological deficits assessment

Grid-walking and foot-fault tests, corner test and neurological deficit score (NDS) were used to assess neurological deficits. For the foot-fault test, on the day before MCAO, and Days 1, 7, 14, and 21 post MCAO, mice were placed in the central square of an apparatus (45 × 45 cm metal grid with grid cells measuring 1 × 1 cm, elevated 50 cm above the floor) and were free to explore for 1 min. A foot fault was recorded whenever a paw slipped. The data are expressed as the percentage of errors made by the contralateral limbs versus the total number of steps. Each animal underwent 3 trials by two investigators who were blinded to the experimental groups. For corner test, two black boards with dimensions of 30 × 20 × 1 cm^3^ were placed together at a 30° angle. On the day before MCAO, on Days 3, 7, 14, and 21 post-MCAO, mice were placed at the middle of the open side and trained to walk toward the corner. When a mouse entered deep into the corner, it would turn backwards to leave the corner. The non-ischemic mouse turned without side preference, whereas the ischemic mouse preferentially turned toward the right side. Ten trials were performed for each mouse and the number of right turns was recorded over the course of ten trials. Neurological deficit score (NDS) was evaluated 1 and 3 d after MCAO. Assessment was performed within 1 min and repeated three times by two investigators who were blinded to the experimental groups. Longa scores were used to determine the motor motion functions: 0, no deficits; 1, difficulty in fully extending the contralateral forelimb; 2, unable to extend the contralateral forelimb; 3, mild circling to the contralateral side; and 4, severe circling. A higher score suggested a more severe neurological deficit.

### Evaluation of infarct size by 2,3,5-triphenyl tetrazolium chloride (TTC) staining

The animals were sacrificed by injecting 60 mg/kg pentobarbital intraperitoneally on Day 3 after MCAO. Infarct size was evaluated by TTC staining. Specifically, the brain tissue was cut into 2-mm-thick pieces and stained with 2% TTC at 37 °C in the dark for 30 min, followed by fixation with 10% formalin at 25 °C overnight. Areas lacking red staining were defined as the infarct area. The infarct area and the whole area of each brain slice were measured using Image J. The infarct volume was calculated by multiplying the added infarct areas of each slice by the slice thickness (2 mm).

### Measurement of brain water content

The mice were sacrificed under deep anesthesia at Day 3 post-MCAO, and the brains were quickly divided into the cerebellum, left hemisphere, and right hemisphere. All tissues were weighed before and after drying for 48 h at 100 °C. Brain water content was subsequently determined using the following equation:brain water content (%) = [(wet weight - dry weight)/wet weight] × 100.

### Immunofluorescence

Mice were subjected to different treatments and were sacrificed with a lethal dose of sodium pentobarbital (0.4 mL) at Day 3 post MCAO. Frozen tissue sections (10 μm thickness) were fixed in 4% paraformaldehyde for 2 h and permeabilized with a 0.1% Triton X-100 solution for 1 h before antigen blocking with 5% goat serum at room temperature for 30 min, followed by incubation with primary antibodies (NeuN 1:200, ab104224, Abcam, USA; caspase-3 1:100, ab197202, Abcam; Iba1 1:1000, Cat#019-19741, Wako, Japan; CD86 1:100, Cat# 91882, Cell Signalling Technology, CST, Hitchin, UK; Arg1 1:200, ab91279, Abcam; Nrf2 1:100, ab31136, Abcam, iNOS, 1:500, ab178945, Abcam) overnight at 4 °C. Subsequently, samples were stained with Alexa Fluor 488 or Alexa Fluor 555-labeled secondary antibody and DAPI in the dark. Finally, the tissue sections were observed under a laser confocal microscope and positive cells were counted using Image J analysis software.

### hUC-MSC tracing in central nervous system (CNS)

The C57BL/6 mice were divided into three groups as follows: (1) sham + hUC-MSCs group, (2) MCAO + hUC-MSCs, and (3) MCAO + hUC-MSCs + curcumin group. Mice that did not receive MCAO operation were defined as the sham group and were transplanted with hUC-MSCs and 0.04 mL of DMSO intraperitoneally. The mice in the MCAO + hUC-MSCs group were transplanted with hUC-MSCs and 0.04 mL of DMSO intraperitoneally post MCAO. The mice in the MCAO + hUC-MSCs + curcumin group were intravenously injected with hUC-MSCs combined with 100 mg/kg curcumin intraperitoneally. 24 h post hUC-MSCs transplantation, mice were sacrificed with a lethal dose of sodium pentobarbital (0.4 mL) and the cerebrum slices were stained with immunofluorescence mentioned above. The hUC-MSCs were identified with anti-human nuclei antibody (1:200, Cat# MAB1281, Millipore), STRO-1 (1:250, Cat#39-8401, Invitrogen) and CD44 (1:500, ab254530, Abcam), respectively. The number of human cells were quantified with Image J.

### Western blotting

Total protein and nucleoprotein (for Nrf2 and β-TrCP) in the ipsilateral peri-infarct hemisphere were extracted, and the protein concentration was determined by bicinchoninic acid protein (BCA) assay. The protein samples were then denatured by boiling for 10 min. Then, 20 μg of each protein sample was separated by sodium dodecyl sulfate polyacrylamide gel electrophoresis (SDS-PAGE) at a constant voltage of 80 V for 100 min. After transfer to polyvinylidene fluoride (PVDF) membrane, the separated protein bands were incubated with individual primary antibodies (caspase-3 1:500, ab197202, Abcam; AKT, 1:1000, Cat# 9272, CST; p-AKT, 1:1000, Cat#9271, CST; GSK-3β, 1:1000, Cat# 9315, CST; p-GSK-3β, 1:1000, Cat# 9336, CST; β-TrCP, 1:1000, Cat# 4394, CST; Nrf2 1:1000, ab92946, Abcam; PCNA, 1:1000, ab92552, Abcam; HO-1, 1:2000, ab52947, Abcam; NQO1, 1:10000, ab80588, Abcam) at 4 °C overnight, followed by incubation with corresponding secondary antibodies at room temperature for 2 h. The developed bands were observed under a gel imaging system.

### Enzyme-linked immunosorbent assay (ELISA)

The left hemisphere was dissected out 3 d after MCAO. After homogenization, the homogenates were centrifuged at 4 °C at 12,000×*g* for 15 min, and supernatants were collected and evaluated in duplicate using IL-1β, TNF-α, IL-6, IL-4, IL-10, TGF-β1, MDA, SOD and GSH-Px ELISA kit (eBioscience, Santiago, USA) in accordance with the manufacturer's guidelines. Briefly, after the standard samples were prepared, the homogenates were incubated at 37 °C for 30 min with the ELISA kits followed by measurement of absorbance at 450 nm in a microplate reader. The concentrations of the samples were calculated from a standard curve.

### Flow cytometric analysis (FACS analysis)

The left hemisphere tissue was mechanically homogenized 3 d after MCAO. Tissue suspensions were incubated in dissociation buffer (10 mL RPMI-1640, 180U collagenase IV, 250U DNase I) for 60 min and overlaid on Percoll gradients of 1.03 and 1.088 g/mL densities. After gradient centrifugation, the collected mononuclear cells were used for flow cytometry staining with CD11b (Clone M1/70, Biolegend, USA), CD45 (Clone 30-F11, Biolegend), CD86 (Clone GL-1, Biolegend), and CD206 (Clone C068C2, BioLegend). We performed multicolor flow cytometry on a FACS Verse and analyzed the data by fluorescence-activated cell sorting (FACS) using Flowjo software. Gates were set according to unstained samples and isotype controls [27].

### Real-time polymerase chain reaction (RT-PCR)

TRIzol reagent was used to extract total RNA from the ipsilateral peri-infarct hemisphere. The purity and concentration of the total RNA were determined and followed by quantitative PCR (TaKaRa) in accordance with the manufacturer's instructions. Primer sequences were purchased from Shanghai Sangon Biological Engineering Technology (Shanghai, China) and are listed in Table 1 [28].

**Table 1:** PCR primers used in this study

	Forward primer	Reverse prime
iNOS	GGCACAGGGTCATCATCAAA	TCAGGTCACTTTGGTAGGATTT
CD86	GTAGAGTCCAGTTGTTCCTGTC	TGGTTCTGTACGAGCACTATTT
arginase-1	GTCCCTAATGACAGCTCCTTT C	CCACACTGACTCTTCCATTCTT
CD206	GTGGTCCTCCTGATTGTGATAG	CACTTGTTCCTGGACTCAGATTA

### Culture of microglia

The cortex of newborn mice was dissected and the cerebral meninges were stripped in HBSS under an anatomical microscope. Tissues were digested in 0.125% trypsin for 8 min and stopped from digestion with 10% FBS followed by filtering through a 200-mesh metal strainer. After centrifugation, a cell suspension (at the density of 1 × 10^6^ cells/mL) was prepared in DMEM/F12 containing 1% antibiotics (100 μg/mL penicillin and 100 μg/mL streptomycin) and 10% FBS, which was placed into an incubator with 5% CO_2_ and incubated at 37 °C for 12 h. Subsequently, the suspension was replaced after the cells adhered to the walls. Thereafter, the culture medium was replaced once every 3 d. A shaker was used to obtain microglia at Day 12, at a rate of 250 rpm for 1 h. The purity of Iba1 positive microglial cells was higher than 95% [26].

### Culture of neurons

Cortical cells were prepared from embryos of pregnant mice at Day 15 [16]. Briefly, after the embryos were removed, the cerebral cortex was dissected, and the meninges were stripped in HBSS under an anatomical microscope. Tissues were then digested in 0.125% trypsin for 8 min and passed through a 40 μm cell strainer. Cells were distributed in a poly (l-lysine)-coated culture plate containing 0.5 mL of neurobasal medium with 2% B27 supplement, at a density of 5 × 10^5^ cells/mL. Cultures were maintained at 37 °C in a humidified incubator with 5% CO_2_. Thereafter, half of the culture medium was replaced once every 2 d. On Day 7, the neurons were harvested and identified by neuronal nuclei (NeuN) staining. The purity of neurons was higher than 95% for following experiments.

### Establishment of oxygen glucose deprivation (OGD)

Oxygen glucose deprivation (OGD) was established as described previously [26]. Briefly, primary microglia were placed into an anaerobic chamber (Thermo, USA), and the culture medium was replaced with glucose-free DMEM/F12 containing 10% FBS. Oxygen in the chamber was replaced with a mixture of 95% N_2_ and 5% CO_2_. After 6 h, the medium was replaced with the original medium in a humidified atmosphere of 5% CO_2_ and 95% air at 37 °C.

### Cell counting kit-8 (CCK-8) detection of curcumin toxicity

Microglia and hUC-MSC were inoculated at the density of 3,000 cells per 96-well plate with different concentrations of curcumin in triplicate for 24 h. The cells were then incubated in 100 μL serum-free medium containing 10 μL CCK-8 (Dojindo Laboratories, Kumamoto, Japan) (i.e., for a total mixture volume of 110 μL) at 37 °C for 2 h, followed by optical density (OD) detection at a wavelength of 450 nm using a microplate reader. Drug toxicity = (treatment group – control group)/(control group – blank group).

### Microglia polarization

Microglia were plated onto the bottom side of the Transwell inserts (0.4 μm pore polyester membrane precoated with PLL) (Corning, USA) at a cell density of 2 × 10^6^ cells/mL followed by OGD for 6 h. The Transwell chambers were positioned approximately 2 mm above the mircoglia-enriched culture plate, and the hUC-MSC (at the cell density of 1 × 10^6^ cells/mL) grown on the Transwell chambers were separated from the microglia by the permeable Transwell membrane. Then 4 μmol/L curcumin (Additional file 1: Fig. S1) was added to the media and incubated for another 6 h. The MK2206, SNP and TDZD-8 were added to the media consisted with curcumin. Cells in the control group were cultured without OGD. Microglia polarization was analyzed by a FACS can flow cytometer and immunofluorescence as mentioned above.

### Analysis of intracellular ROS levels

Primary microglia were treated with serum-free medium for 1 h, followed by OGD. After that, the microglia were subjected polarized incubated by hUC-MSC with or without curcumin, MK2206, SNP and TDZD-8 interventions. At 6 h after treatment, DCF-DA (a fluorescent probe) was used to detect the intracellular ROS levels using a commercially available kit. Briefly, cells were incubated with 10 μM DCF-DA at 37 °C for 20 min and then the fluorescence of DCF was observed by confocal microscopy or quantified at 525 nm after excitation at 488 nm.

### Neuron apoptosis

After the aforesaid polarization procedure, the media were replaced and the microglia were cultured with serum-free DMEM/F12 medium for 6 h. Then the supernatant was collected and supplemented with 2% B27 for neurobasal medium (conditioned medium, CM). The primary neurons were inoculated at the density of 2 × 10^6^ cells/mL with OGD for 6 h, followed by a replacement with the CM for 6 h. Immunofluorescence and Annexin V-FITC Apoptosis Detection Kit was used to analyze neuron apoptosis. In brief, neurons were collected with StemPro Accutase Cell Dissociation Reagent (Life Technologies, A1110501). Cells were incubated with Annexin V-FITC at room temperature for 10 min and resuspended in the binding buffer. Then propidium iodide (PI) was added into the obtained suspension and incubated for 5 min on ice. Cells were analyzed using a FACScan flow cytometer.

### Statistical analysis

All data are presented as mean ± standard deviation (SD). SPSS22.0 software (SPSS Inc., Chicago, IL, USA) was used for statistical analysis. One-way ANOVA with LSD’s post-hoc tests were used to analyze statistical significance except the forelimb fault rate and the right turns rate data. Two-way repeated-measures ANOVA with LSD’s post-hoc tests were used to analyze the data for the behavior assessment of the forelimb fault rate and the right turns rate only. P < 0.05 was considered statistically significant.

## Results

### Characterization of and tracing hUC-MSCs

Flow cytometry was used to characterize hUC-MSCs. The hUC-MSC markers such as CD73 (99.9%), CD90 (98.6%), and CD105 (100%) were highly expressed, in parallel with low expression of hematopoietic stem cell markers such as CD34 (0.3%), CD45 (0.1%), and HLA-DR (0.0%) (Additional file 1: Fig. S2). These data confirmed the correct identity of hUC-MSCs, and the purity of these cells met the standard required for subsequent experiments. Immunofluorescence was used to tracing the migration of the hUC-MSCs in the CNS (Additional file 1: Fig. S3A). The green and red fluorescence was not detected in the sham group declared that hUC-MSCs could not traverse the normal blood brain barrier (BBB) in the physiological circumstances. In contrast, hUC-MSCs appeared in the infarct area in the MCAO group. These data demonstrated that hUC-MSCs could migrate to the CNS due to the increased permeability of the BBB. Furthermore, compared with the MCAO group, there were more hUC-MSCs detected in the MCAO + hUC-MSCs + curcumin group, indicating curcumin could enhance the hUC-MSCs survival in the CNS (Additional file1: Fig. S3B–D).

### Combined curcumin-hUC-MSC treatment improved functional performance outcomes after MCAO

The therapeutic effects of combined curcumin-hUC-MSC treatment were examined in MCAO mice. From Day 1 to Day 21 post-MCAO, the forelimb fault rate (Fig. 1D) and the right turns rate (Fig. 1E) of mice gradually decreased, indicating that motor function could recover spontaneously. There were no significant differences in the functional performance outcomes between the MCAO + hUC-MSCs and MCAO + curcumin groups at 21 d post-MCAO, demonstrating that the effect of hUC-MSCs on the motor function recovery was analogous to curcumin treatment. However, the MCAO + hUC-MSCs + curcumin group registered improved neurological deficit at each time point compared with that of the MCAO + hUC-MSCs and MCAO + curcumin groups.

TTC staining (Fig. 1F) revealed that the normal brain tissues stained red, and the ischemia injured brain displayed a pale color. There was no statistical difference in the infarct volume between MCAO + hUC-MSCs and MCAO + curcumin groups. Significant differences were observed in the infarct volume among the MCAO (64.86 ± 7.95 mm^3^), MCAO + hUC-MSCs (37.51 ± 5.55 mm^3^) and the MCAO + hUC-MSCs + curcumin (24.33 ± 5.31 mm^3^) groups (Fig. 1G). MCAO led to cerebral edema and increased brain water content in both the ipsilateral and contralateral areas of the infarct and cerebellum. We found that the combined hUC-MSCs and curcumin treatment decreased brain water content (Fig. 1H), particularly in the ipsilateral region of the infarct and this decrease was statistically significant when compared with MCAO + hUC-MSCs and MCAO + curcumin groups. Together, these data revealed that the combined curcumin-hUC-MSC treatment could improve outcomes on functional tests and diminish infarct volume during recovery post-MCAO.

### Combined curcumin-hUC-MSC treatment decreased neuronal apoptosis

**Fig. 2:**
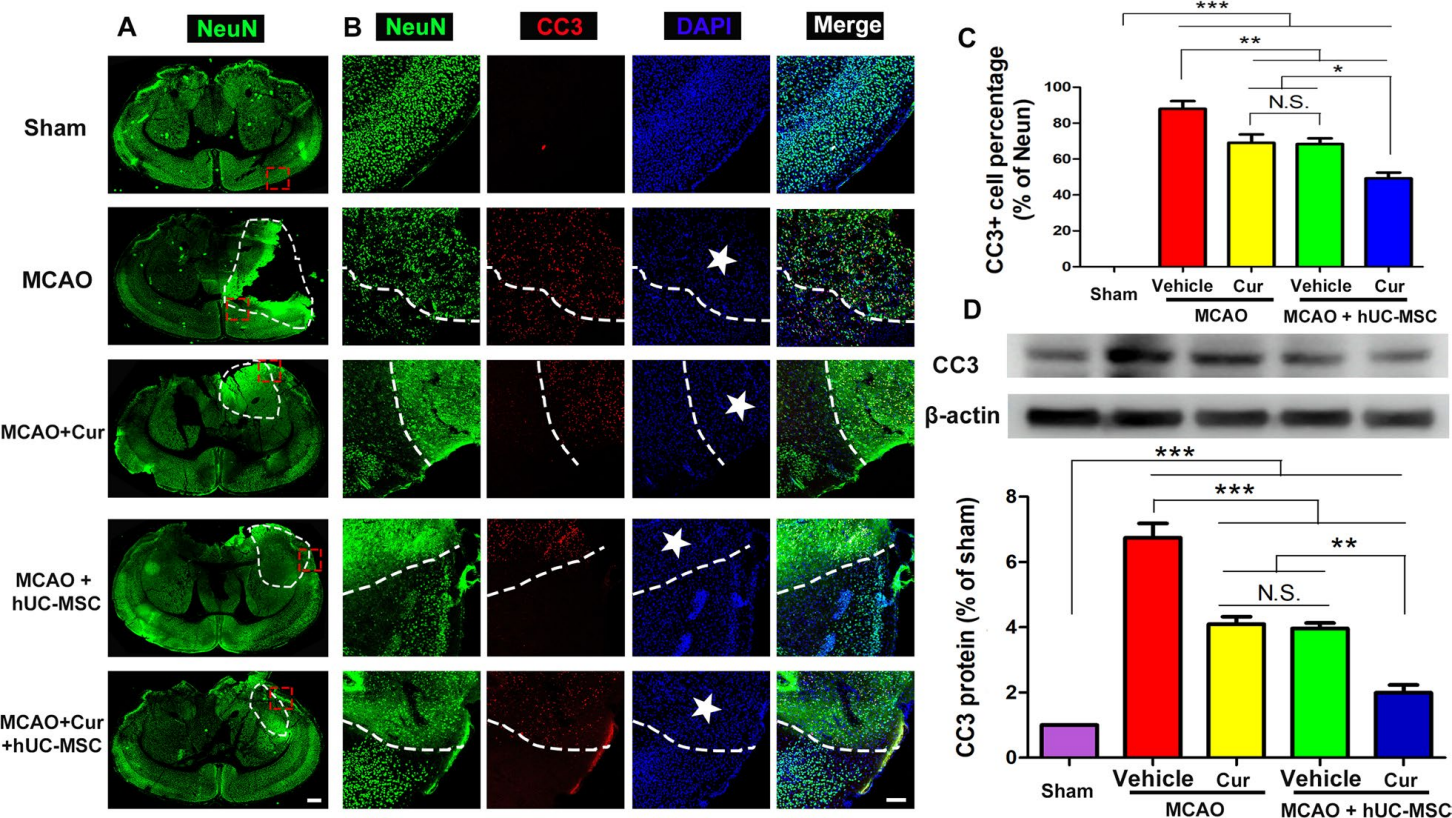
Combined curcumin-hUC-MSC treatment decreased neuronal apoptosis. **A** Representative images of immunofluorescence analysis for neuronal apoptosis with whole brain tissue, scale bars = 600 μm. **B**, **C** Representative images (**B**) and quantification (**C**) of immunofluorescence analysis for neuronal apoptosis in the ischemic penumbra. The neuron-specific marker NeuN (green), and the apoptosis marker CC3 (red). Nuclei were stained with DAPI (blue). The white five-pointed star indicates the infarct core, scale bar, 20 μm. **D** Western blot and quantitative analysis of the expression of CC3 in the infarction region of the ipsilateral cerebral hemisphere. *Cur* curcumin, N = 5; *N.S.* no significant difference, *P < 0.05, **P < 0.01, ***P < 0.001

Neuronal apoptosis caused by cerebral ischemia is a fundamental factor leading to motor dysfunction. We observed that post MCAO, the NeuN^+^ cells decreased with an increase in CC3^+^ cells (Fig. 2A, B). Moreover, compared with MCAO group, the number of NeuN^+^ cells in MCAO + hUC-MSCs and MCAO + curcumin groups were higher, while the number of CC3^+^ cells were lower. Combined hUC-MSCs and curcumin treatment showed a reduction in the number of CC3^+^ cells suggesting that this treatment protected neurons from apoptosis. Western blot analysis (Fig. 2C, D) showed an increased level of CC3 in the infarcted cerebral hemisphere of the MCAO group. The hUC-MSC transplantation and curcumin treatment notably reduced CC3 levels, especially in the combined hUC-MSCs and curcumin treatment. These results substantiate that combined curcumin- hUC-MSC treatment promoted functional recovery by suppressing neuronal apoptosis.

### Combined curcumin-hUC-MSC treatment alleviated inflammation and oxidative stress in MCAO mice

**Fig. 3:**
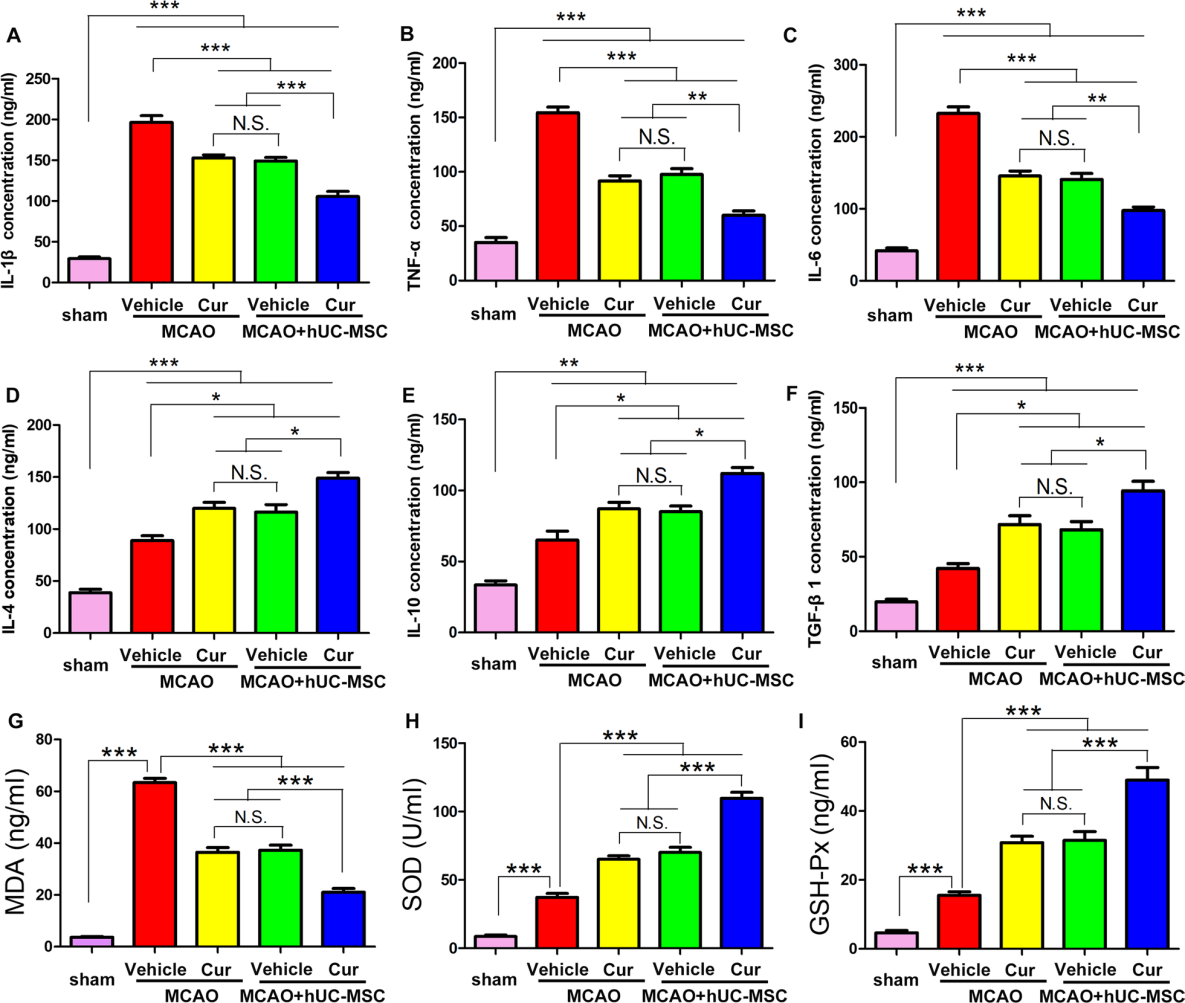
Combined curcumin-hUC-MSC treatment alleviated inflammation and oxidative stress response in MCAO mice. **A**–**C** The expression levels of pro-inflammatory cytokines IL-1β (**A**); TNF-α (**B**); and IL-6 (**C**) in the infarct region of the ipsilateral cerebral hemisphere; (**D**–**F**) the expression levels of anti-inflammatory cytokines IL-4 (**D**); IL-10 (**E**); and TGFβ1 (**F**) in the infarct region of the ipsilateral cerebral hemisphere. **G**–**I** The expression levels of markers of oxidative damage, MDA (**G**), SOD (**H**), and GPx (**I**) in the infarct region of the ipsilateral cerebral hemisphere with different treatments. *Cur* curcumin, N = 5; *N.S.* no significant difference, *P < 0.05, **P < 0.01, ***P < 0.001

Inflammation and oxidative stress response are the main causes of neuronal apoptosis [26]. Therefore, we next investigated the inflammatory cytokines and antioxidative activities in the hUC-MSCs transplantation and curcumin treatment in MCAO mice (Fig. 3). Compared with the MCAO group, the typical pro-inflammatory cytokines, such as interleukin (IL)-1β, tumor necrosis factor (TNF)-α, and IL-6 decreased in MCAO-hUC-MSCs and MCAO + curcumin groups. Concomitantly an increase in the typical anti-inflammatory cytokines, such as IL-4, IL-10, and transforming growth factor (TGF) β1 was observed. MCAO + hUC-MSCs + curcumin showed a further decrease in the pro-inflammatory cytokines and an augmentation of the anti-inflammatory cytokines. Furthermore, the expression trends for the typical oxidative mediators (including MDA, SOD, and GPx) were consistent with pro-inflammatory cytokines. There is no statistical difference for the expression of pro- and anti-inflammatory cytokines and antioxidative activities between MCAO-hUC-MSCs and MCAO + curcumin groups. The pro-inflammatory cytokines and antioxidative activities are detrimental and anti-inflammatory cytokines are beneficial for tissue restoration. Collectively, these data demonstrate that the combined curcumin-hUC-MSC treatment alleviated inflammation and oxidative stress which likely provided a neuroprotective effect in MCAO mice.

### Combined curcumin-hUC-MSC treatment promotes anti-inflammatory phenotype microglia in MCAO mice

**Fig. 4:**
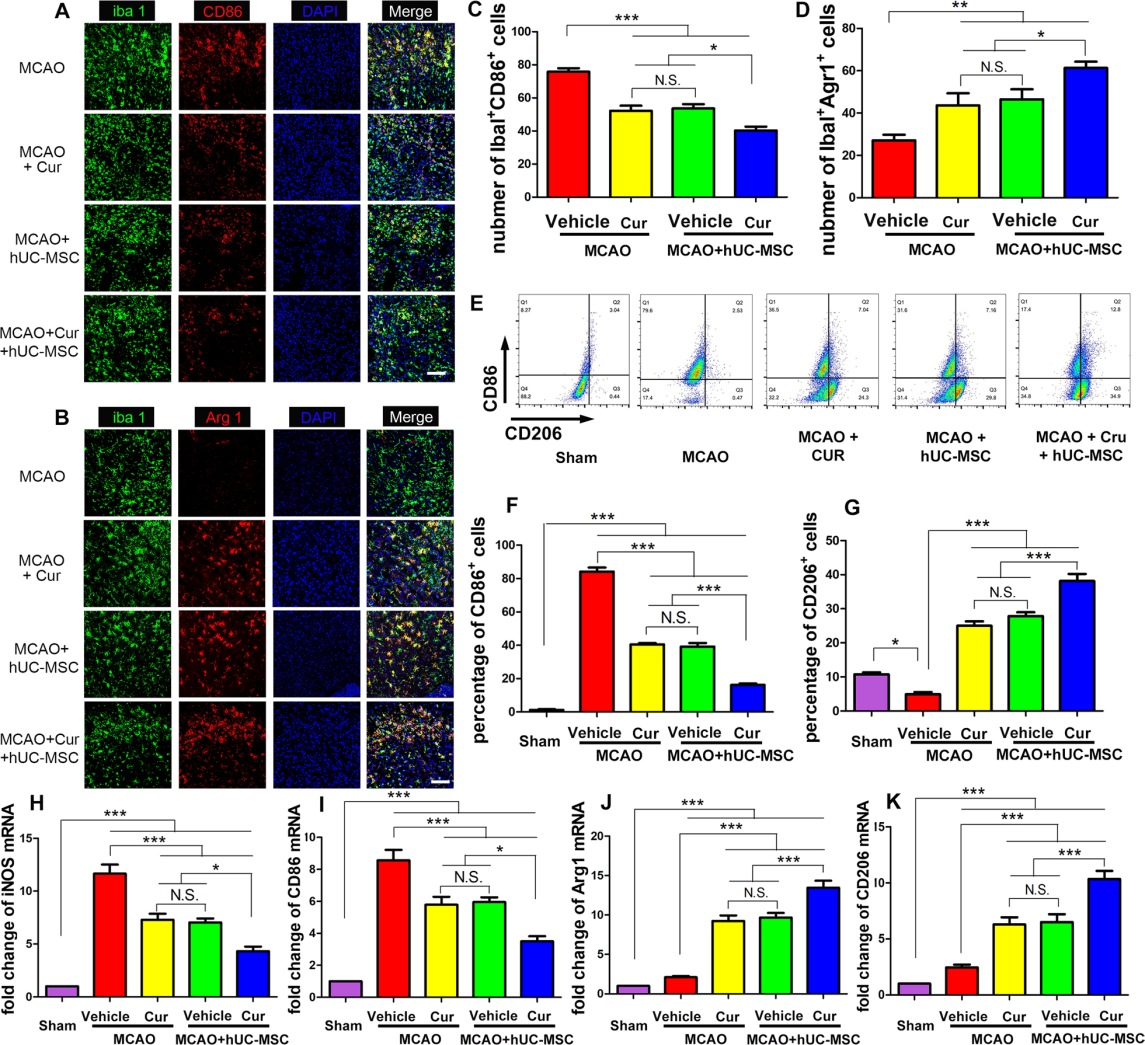
Combined curcumin-hUC-MSC treatment promoted anti-inflammatory phenotype microglia in MCAO mice. **A**, **B** Representative images of immunofluorescence analysis for microglia polarization, (**A**) The microglial cell-specific marker Iba1 (green), pro-inflammatory microglia marker CD86 (red), nuclei (blue); (**B**) the microglial cell-specific marker Iba1 (green), anti-inflammatory microglia marker Arg1 (red), nuclei (blue). **C**, **D** Quantitative analysis of pro-inflammatory (**C**) and anti-inflammatory (**D**) microglia in the ipsilateral peri-infarct. **E** Representative images of flow cytometry analysis for microglia polarization. **F**–**G** Quantitative analysis of the percentage of CD86^+^ cells (**F**) and CD206^+^ microglia (**G**) in the ipsilateral peri-infarct isolated from MCAO mice subjected to different treatments. **H**, **I** qRT-PCR analysis for iNOS (**H**) and CD86 (**I**) related to pro-inflammatory microglia gene and Arg1 (**J**) and CD206 (**K**) related to anti-inflammatory microglia gene. *Cur* curcumin, scale bars = 20 μm; N = 5; *N.S.* no significant difference, *P < 0.05, **P < 0.01, ***P < 0.001

Microglia are resident immune cells of the central nervous system, which secrete the pro-inflammatory and anti-inflammatory cytokines to mediate local inflammation during ischemic stroke. We found that the anti-inflammatory cytokines were increased, and the pro-inflammatory cytokines were decreased after combined treatment in MCAO mice. To assess the mechanisms underlying the inhibition of inflammation mediated by the combined curcumin-hUC-MSCs treatment, we examined the phenotypic polarization of microglia in MCAO mice (Fig. 4). While MCAO mediated ischemia effectively increased the number of the Iba1^+^CD86^+^ cells, the hUC-MSC and curcumin treatment significantly decreased these pro-inflammatory phenotypes. In contrast, while MCAO mediated ischemia lowered the number of Iba1^+^Arg1^+^ cells, the hUC-MSCs and curcumin combined treatment effectively aggrandized these anti-inflammatory phenotypes. The number of anti-inflammatory phenotype microglia in the MCAO + hUC-MSCs + curcumin group were statistically higher than that in the MCAO + hUC-MSCs and MCAO + curcumin groups (Fig. 4A–D; Additional file 1: Fig. S4). Likewise, a similar trend was revealed in both FACS (Fig. 4E–G) and mRNA analyses (Fig. 4H–K). These data demonstrate that the combined of curcumin and hUC-MSC treatment promotes microglial polarization to the anti-inflammatory phenotype and attenuates the pro-inflammatory phenotype in MCAO mice.

### Combined curcumin-hUC-MSC treatment mediated the Nrf2 expression

**Fig. 5:**
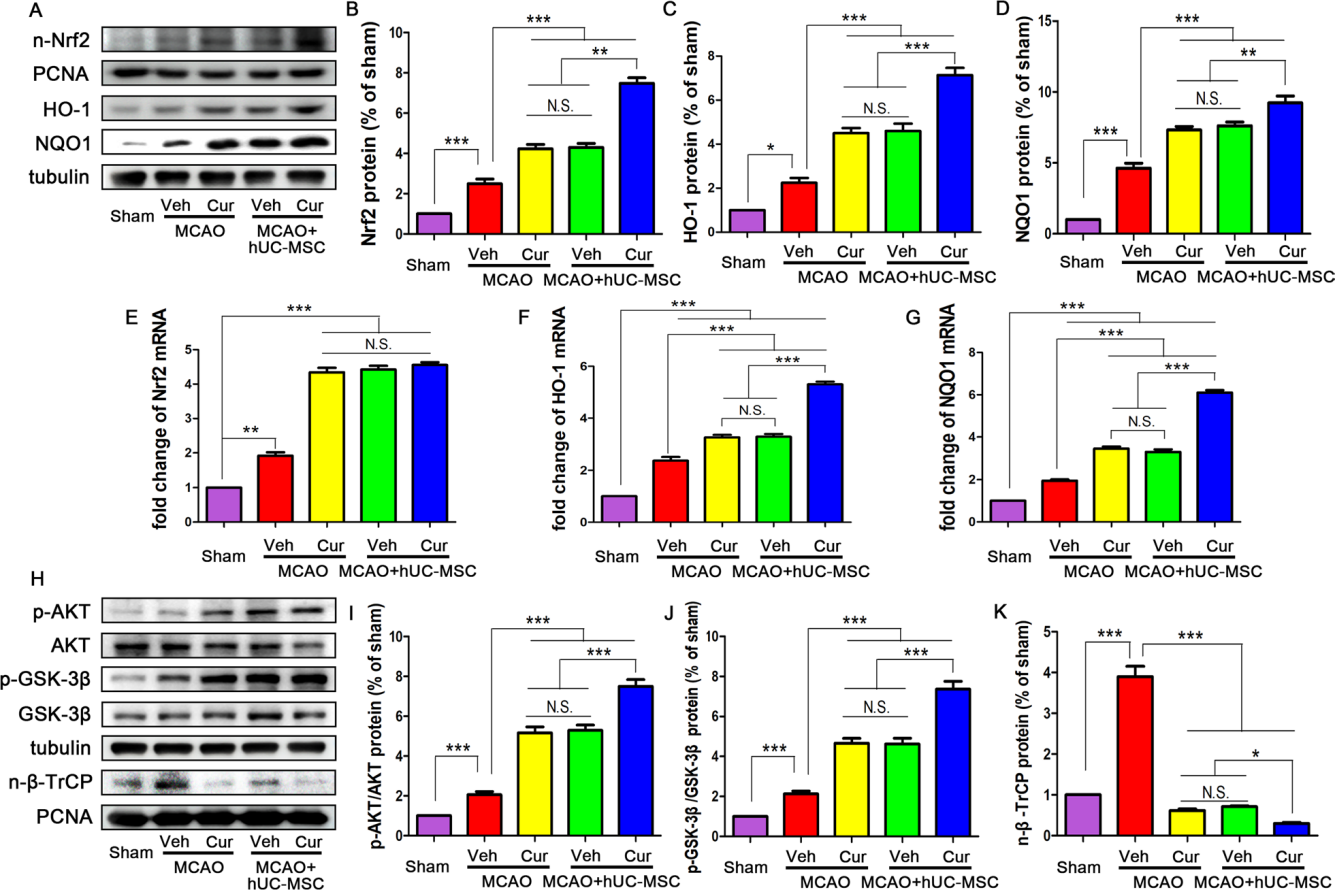
AKT/GSK-3β/β-TrCP/Nrf2 pathway was involved in the combined treatment for the MCAO in vivo. **A**–**D** Representative western blots (**A**) and quantitative data for the expression of Nrf2 in the nucleus (**B**) and the downstream effectors of Nrf2 pathway including HO-1 (**C**) and NQO1 (**D**). The hUC-MSCs transplantation and curcumin treatment significantly increased the expression of nuclear Nrf2, HO-1 and NQO1. The expression of Nrf2, HO-1 and NQO1 in MCAO + hUC-MSCs + curcumin group was significantly higher than that in the other groups. **E**–**G** qRT-PCR analysis for Nrf2 (**E**), HO-1 (**F**) and NQO1 (**G**) with different treatments. There were no differences among the MCAO + hUC-MSCs, MCAO + curcumin and the MCAO + hUC-MSCs + curcumin groups in Nrf2 mRNA expression, while combined treatment upregulated the HO-1 and NQO1 expression. **H**–**K** Representative western blots (**H**) and quantitative data for the AKT (Ser473) (**I**), GSK-3β (Ser9) (**J**), and β-TrCP in the nucleus (**K**) for the proteins upstream of the Nrf2 pathway. Combined treatment increased the expression of p-AKT (Ser473), p-GSK-3β (Ser9) and suppressed the nuclear β-TrCP expression. *Cur* curcumin, N = 5; *N.S.* no significant difference, *P < 0.05, **P < 0.01, ***P < 0.001

Nuclear factor erythroid 2-related factor 2 (Nrf2) is a major regulator of antioxidant and anti-inflammatory response. In the ischemic brain, an increase in Nrf2 protein expression concomitant with the expression of the downstream proteins HO-1 and NQO1 and reduced the inflammatory response were observed (Fig. 5A–D). The hUC-MSCs and curcumin treatment significantly increased the expression of Nrf2, HO-1 and NQO1 protein, indicating that hUC-MSCs and curcumin possessed potential anti-inflammatory and antioxidant effect. Compared with the MCAO + hUC-MSCs and MCAO + curcumin groups, the levels of Nrf2, HO-1 and NQO1 in the MCAO + hUC-MSCs + curcumin were higher. Additionally, the mRNA expression of the Nrf2, HO-1 and NQO1 were up-regulated with curcumin and hUC-MSC treatment when compared with the MCAO group. Interestingly, there were no significant difference among the MCAO + hUC-MSCs, MCAO + curcumin and the MCAO + hUC-MSCs + curcumin groups in Nrf2 mRNA expression, while the combined treatment upregulated the HO-1 and NQO1 expression (Fig. 5E–G). These data indicate that combined curcumin-hUC-MSC treatment enhanced nuclear accumulation and stabilization of Nrf2 without affecting its transcription. Collectively, these results demonstrated that the combination of curcumin and hUC-MSC transplantation could reinforce the expression of Nrf2 pathway.

### AKT/GSK-3β/β-TrCP axis was involved in the Nrf2-mediated antioxidant and anti-neuroinflammatory activity in MCAO mice with combined treatment

As the combined curcumin-hUC-MSC treatment increased the Nrf2 expression in the protein level without altering in the mRNA level when compared with other treatments, we speculated that the combined treatment might be suppressing the degradation of Nrf2 exported from the nucleus to the cytoplasm. Several studies have certified the role of AKT/GSK3β/β-TrCP axis in the regulation of Nrf2 nuclear export. Activated β-TrCP kinase could translocate into the nucleus to degrade the Nrf2 by exporting it from the nucleus to the cytoplasm. Moreover, the AKT phosphorylation (activation) could promote GSK3β phosphorylation (inactivation) resulting in an inhibition of the β-TrCP expression [29, 30]. Therefore, we explored the role of AKT/GSK3β/β-TrCP axis in Nrf2 nuclear accumulation in response to the combined treatment. The WB results showed that (Fig. 5H–K) when compared with the MCAO group, single curcumin treatment or hUC-MSC transplantation could increase the AKT and GSK3β phosphorylation, accompanied with suppression the of the β-TrCP nuclear translocation. Combined curcumin-hUC-MSC treatment significantly enhanced the activation by AKT phosphorylation and GSK3β inactivation and inhibited the expression of the β-TrCP in the nucleus. These data indicate that AKT/GSK-3β/β-TrCP axis was involved in the Nrf2-mediated antioxidant and anti-neuroinflammatory activity in response to the combined treatment in MCAO mice.

### Combined curcumin-hUC-MSC treatment enhanced the Nrf2 nuclear accumulation through AKT/GSK-3β/β-TrCP axis in microglia

**Fig. 6:**
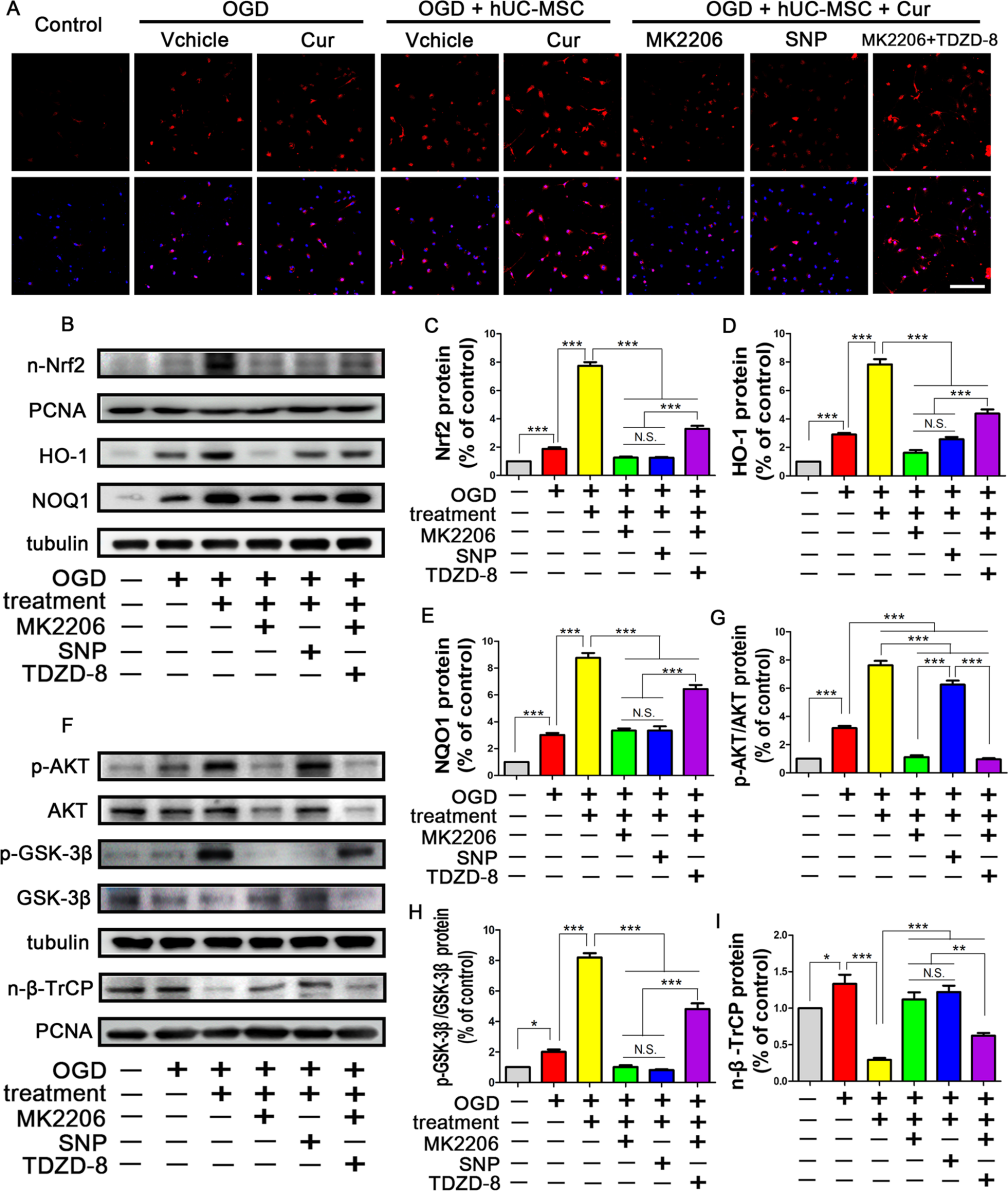
Combined curcumin and hUC-MSCs treatment activated the AKT/GSK-3β/β-TrCP/Nrf2 axis in microglia in vitro. **A** Representative images of immunofluorescence analysis of Nrf2 expression in microglia treated with the AKT inhibitor MK2206, the GSK3β activator sodium nitroprusside (SNP) (a GSK3β phosphorylation inhibitor) and the GSK3β inhibitor TDZD-8. Nrf2 (red), nuclei (blue). **B**–**E** Representative western blots (**B**) and quantitative data for the expression of Nrf2 in the nucleus (**C**) and the downstream proteins of Nrf2 pathway including HO-1 (**D**) and NQO1 (**E**) with different treatments. **F**–**I** Representative western blots (**F**) and quantitative data for the AKT (Ser473) (**G**), GSK-3β (Ser9) (**H**), and β-TrCP in the nucleus (**I**) related for the upstream proteins of Nrf2 pathway. *Cur* curcumin, scale bars = 20 μm; N = 5; *N.S.* no significant difference, *P < 0.05, **P < 0.01, ***P < 0.001

We next explored the Nrf2 activation in microglia in vitro post-OGD treated with curcumin or hUC-MSC. The results (Fig. 6) revealed that the proteins of downstream the Nrf2 pathway including HO-1 and NQO1 were up-regulated suggesting that Nrf2 was activated in the microglial nucleus. Curcumin or hUC-MSC treatment further increased the expression of the nuclear Nrf2. Nrf2 accumulated significantly more in the microglia nuclei with combined treatment when compared with single treatment, consistent with the data from MACO mice (Fig. 6A–E). In order to clarify whether the AKT/GSK-3β/Β-TrCP axis is the pivotal pathway for Nrf2 nuclear retention with curcumin and hUC-MSC treatment, we detected the expression of Nrf2 with in the presence of the AKT inhibitor MK2206, the GSK3β activator sodium nitroprusside (SNP) and a GSK3β inhibitor TDZD-8 (Fig. 6F–I). Compared with the combined treatment group, MK2206 activated GSK3β (inhibited GSK3β phosphorylation) and β-TrCP translocated to the nuclear, which in turn promoted Nrf2 nuclear export and cytoplasmic degradation. Moreover, SNP-mediated activation of GSK3β increased the β-TrCP expression in the nucleus and resulted in reduced levels of nuclear Nrf2 in microglia and suppressed the downstream proteins for HO-1 and NQO1, whereas the GSK3β phosphorylation agonist TDZD-8 exerted the opposite effect. These data demonstrate that combined curcumin-hUC-MSC treatment enhanced nuclear accumulation of Nrf2 through the AKT/GSK-3β/β-TrCP axis in microglia.

### Combined curcumin-hUC-MSC treatment suppressed inflammation and oxidative stress through AKT/GSK-3β/β-TrCP/Nrf2 axis in microglia

**Fig. 7:**
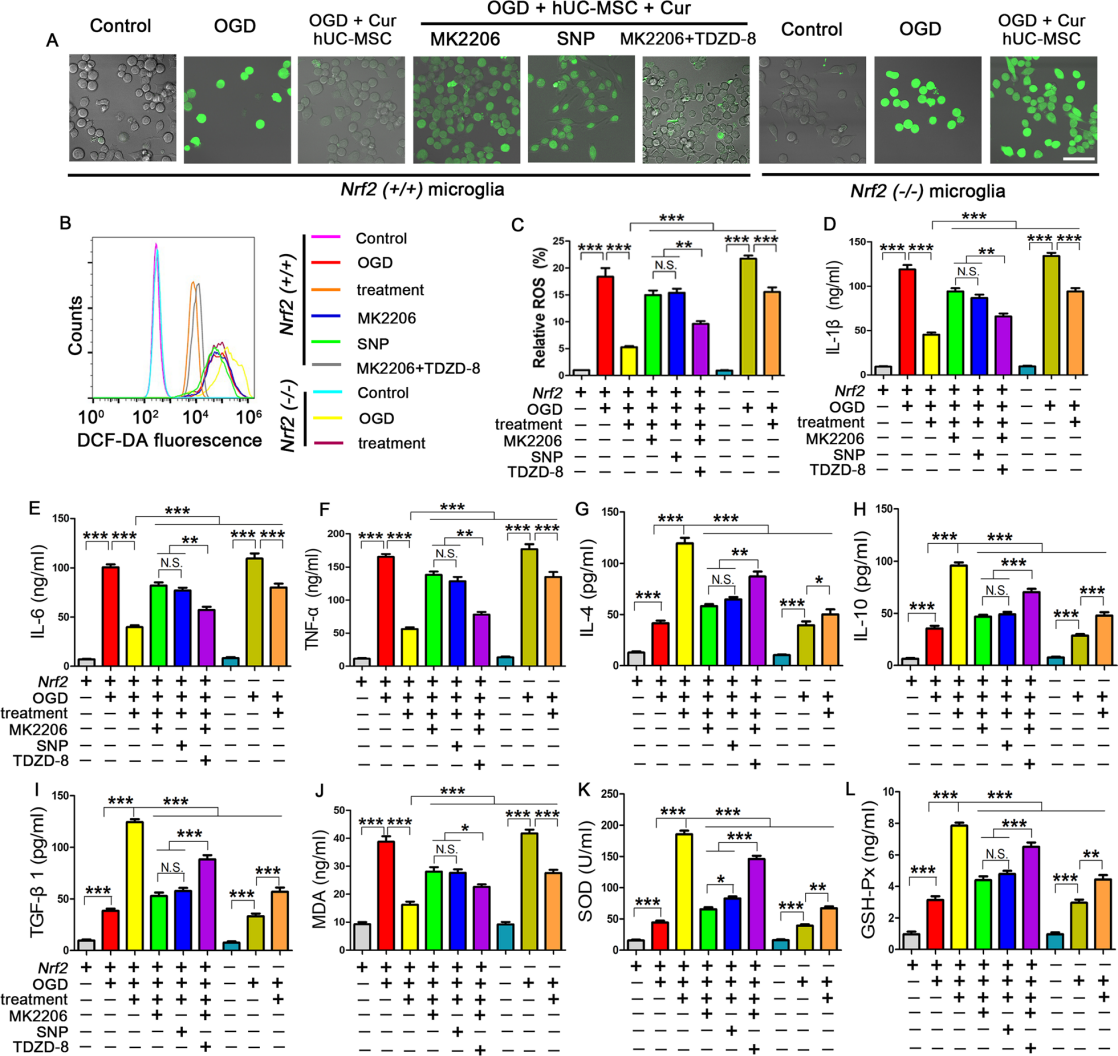
Combined curcumin and hUC-MSCs treatment suppressed ROS generation and exerted antioxidative and anti-inflammatory effects through AKT/GSK-3β/β-TrCP/Nrf2 pathway in vitro. **A** Confocal microscopy images showing relative intracellular ROS levels in microglia. **B**, **C** Flow cytometric curves (**B**) and quantification (**C**) showing DCF-DA fluorescence intensities in microglia. **D**–**I** Quantitative analysis of the expression of pro-inflammatory cytokines IL-1β (**D**), IL-6 (**E**), TNF-α (**F**) and anti-inflammatory cytokines IL-4 (**G**), IL-10 (**H**), TGFβ1 (**I**) in microglia. **J**–**L** The expression levels of oxidative damage-related biomarkers of MDA (**J**), SOD (**K**), and GPx (**L**) in microglia. *Cur* curcumin, scale bars = 20 μm; N = 5; *N.S.* no significant difference, *P < 0.05, **P < 0.01, ***P < 0.001

To assess whether the AKT/GSK-3β/β-TrCP/Nrf2 axis mediated the anti-oxidative stress and anti-inflammation effects of the combined treatment in vitro, we examined the production of ROS, typical oxidative mediators, and inflammatory cytokines in primary microglia subjected to different treatments (Fig. 7). Fluorescence microscopy revealed that the DCF-DA fluorescent signal was more notable in the OGD group than that in the control group, while the combined treatment decreased the DCF-DA fluorescent signal, indicated OGD induced ROS production in microglia and combined treatment inhibited the ROS generation. Compared to single treatment, inhibition of AKT with MK2206 or activation of GSK3β with SNP after combined treatment increased the ROS production in microglia, whereas suppression of the GSK3β after AKT inhibition (MK2206 + TDZD-8) reduced the ROS generation (Fig. 7A–C). In addition, the ELISA results demonstrated that the expression trends of the pro-inflammatory cytokines (IL-1β, IL-6, TNF-α) (Fig. 7D–F) and typical oxidative stress mediators (MDA, SOD, and GPx) (Fig. 7J–L) were consistent with the ROS production, whereas the anti-inflammatory cytokines (IL-4, IL-10, TGFβ1) (Fig. 7G–I) showed the opposite trend. However, *Nrf2* deficiency blocked the beneficial effect of combined treatment on the oxidative stress and inflammation, which is similar to the effects observed when treated with the AKT inhibitor. Intriguingly, we found that *Nrf2* deficiency partly abrogated the combined treatment mediated anti-oxidative stress and anti-inflammation effects when compared with the *Nrf2 *(+*/*+) microglia, indicating that there are still other mechanisms for the combined hUC-MSC transplantation with curcumin treatment besides Nrf2 pathway. Collectively, the above data demonstrate that combined treatment suppressed the oxidative stress and inflammation through AKT/GSK-3β/β-TrCP/NRF2 axis in OGD microglia.

### AKT/GSK3β/β-TrCP/Nrf2 signaling was responsible for anti-inflammatory phenotype microglia polarization mediated by combined curcumin-hUC-MSC treatment

**Fig. 8:**
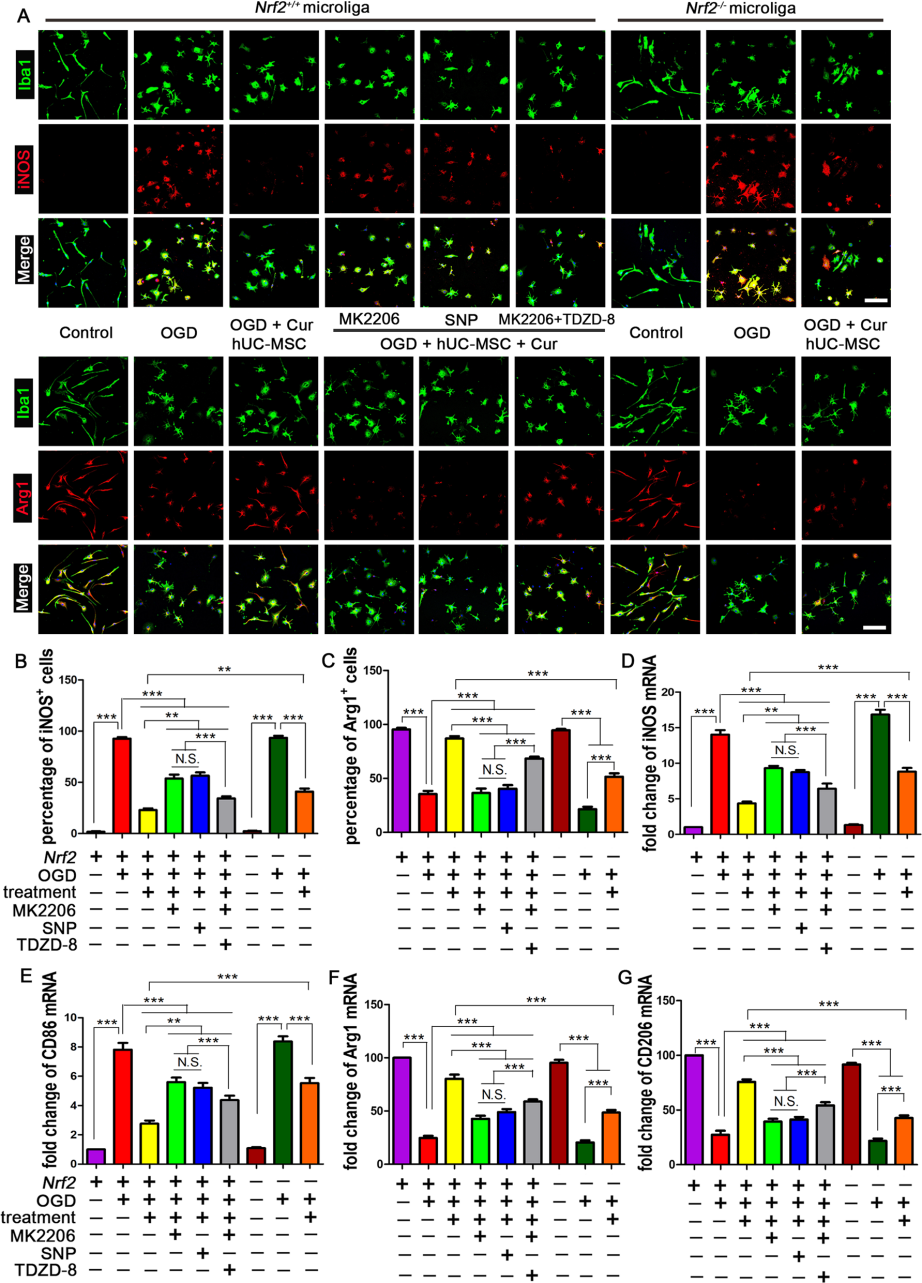
Combined curcumin and hUC-MSCs treatment promoted anti-inflammatory phenotype microglia polarization through AKT/GSK-3β/β-TrCP/Nrf2 pathway in vitro. **A** Representative images of immunofluorescent staining of classically activated pro-inflammatory microglia (upper) and alternatively activated anti-inflammatory phenotype (lower) of the microglia. The microglial cell-specific marker Iba1 (green), pro-inflammatory microglia marker iNOS (upper) or anti-inflammatory microglia marker Arg1 (lower) (red), and nuclei (blue). **B**, **C** Quantitative immunofluorescence of pro-inflammatory with CD86 (**B**) and anti-inflammatory with CD206 (**C**) microglia. **D**–**G** qRT-PCR analysis of iNOS (**D**) and CD86 (**E**) expression for pro-inflammatory phenotype and Arg1 (**F**) and CD206 (**G**) expression for anti-inflammatory phenotype. *Cur* curcumin, scale bars = 20 μm; N = 5; *N.S.* no significant difference, **P < 0.01, ***P < 0.001

Furthermore, we evaluated the effect of AKT/GSK3β/β-TrCP/Nrf2 signaling on microglia phenotypic polarization. In an in vitro experiment, the microglia in the resting state exhibited long and spindle-like morphology and rarely expressed the pro-inflammatory subtype markers, CD86 and iNOS, but mainly expressed the anti-inflammatory subtype markers, CD206 and Arg1 (Fig. 8). With the OGD intervention, the microglia became round and the number of microglia expressing CD86 and iNOS was significantly increased, while the expression levels of CD206 and Arg1 were low (Fig. 8A). Combined curcumin-hUC-MSC treatment with OGD, the microglia suppressed the expression of pro-inflammatory markers and increased that of anti-inflammatory markers at both protein (Fig. 8B–C) and mRNA levels (Fig. 8D–G), demonstrating that combined treatment promoted microglia polarized to anti-inflammatory phenotype. After combined treatment, inhibition of AKT with MK2206 or activation GSK3β with SNP increased pro-inflammatory subtype marker expression and decreased the anti-inflammatory markers, whereas inactivating GSK3β following AKT inhibition (MK2206 + TDZD-8) enhanced anti-inflammatory marker expression. By contrast, *Nrf2* deficiency attenuated CD206 and Arg1 expression and upregulated CD86 and iNOS. Consistent with the previous in vitro experiment, we propose that activated AKT phosphorylation could facilitate GSK3β phosphorylation (inactivation) and inhibit β-TrCP phosphorylation to the nuclear to suppress Nrf2 nuclear export and cytoplasmic degradation, which in turn promoted microglia polarized to anti-inflammatory phenotype after MCAO (Additional file 1: Fig. S5). These data confirm that AKT/GSK3β/β-TrCP /Nrf2 signaling was the critical pathway in the combined hUC-MSCs and curcumin treatment-mediated microglia phenotypic polarization.

### Combined curcumin-hUC-MSC treatment suppressed neuronal apoptosis by microglia paracrine through AKT/GSK-3β/β-TrCP/Nrf2 axis

**Fig. 9:**
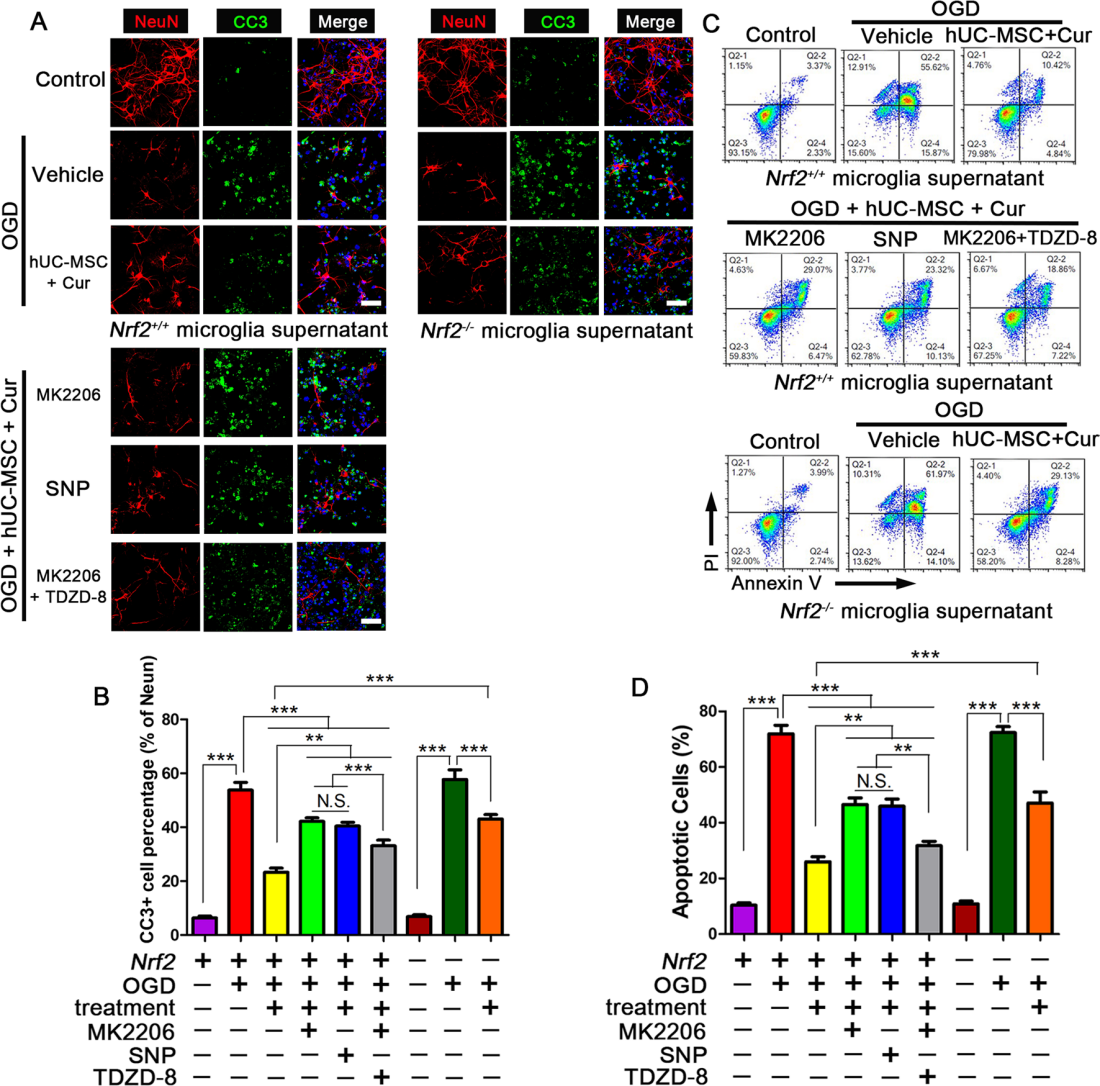
Combined curcumin-hUC-MSC treatment inhibited the neuronal apoptosis by paracrine path via AKT/GSK-3β/β-TrCP/Nrf2 axis in vitro. Collected the supernatant with different treatments in *Nrf2* ^+*/*+^ or *Nrf2 *^*−/−*^ microglia to cultivate the OGD neurons. (A-B) Representative images (**A**) and quantification (**B**) of immunofluorescence analysis of neuronal apoptosis. The neuron-specific marker NeuN (red), the apoptosis marker CC3 (green), and nuclei (blue). **C**, **D** Flow cytometric analysis (**C**) and the quantification (**D**) for the neuronal apoptosis. Cur: curcumin, scale bars = 20 μm; N = 5; *N.S.* no significant difference, **P < 0.01, ***P < 0.001

Next, we cultivated the OGD neurons with the microglia supernatants collected after different treatments to assess the effect of the combined treatment on apoptosis (Fig. 9). Immunofluorescence (Fig. 9A, B) and FACS (Fig. 9C, D) showed that the OGD treatment resulted in significantly higher expression of CC3 compared to the control neurons treated with fresh medium. The expression of CC3 was notably reduced after incubation with supernatant from the combined curcumin and hUC-MSC treatment. Moreover, there were more CC3-positive cells and fewer NeuN-positive cells in the combination treatment in *Nrf2* gene deficient microglia, indicating that the combined treatment suppressed neuronal apoptosis through Nrf2 signaling. Furthermore, we found that the supernatants from the AKT phosphorylation inhibitor MK2206 and GSK3β phosphorylation activator groups increased the number of CC3^+^ neurons, whereas the supernatant from the GSK-3β inhibitor TDZD-8 treatment decreased the number of CC3^+^ neurons. These data demonstrate that combined curcumin-hUC-MSC treatment suppressed neuron apoptosis by microglia through AKT/GSK-3β/β-TrCP/Nrf2 axis.

### *Nrf2* deficiency abolished neurological function improvement induced by combined curcumin-hUC-MSC treatment

**Fig. 10:**
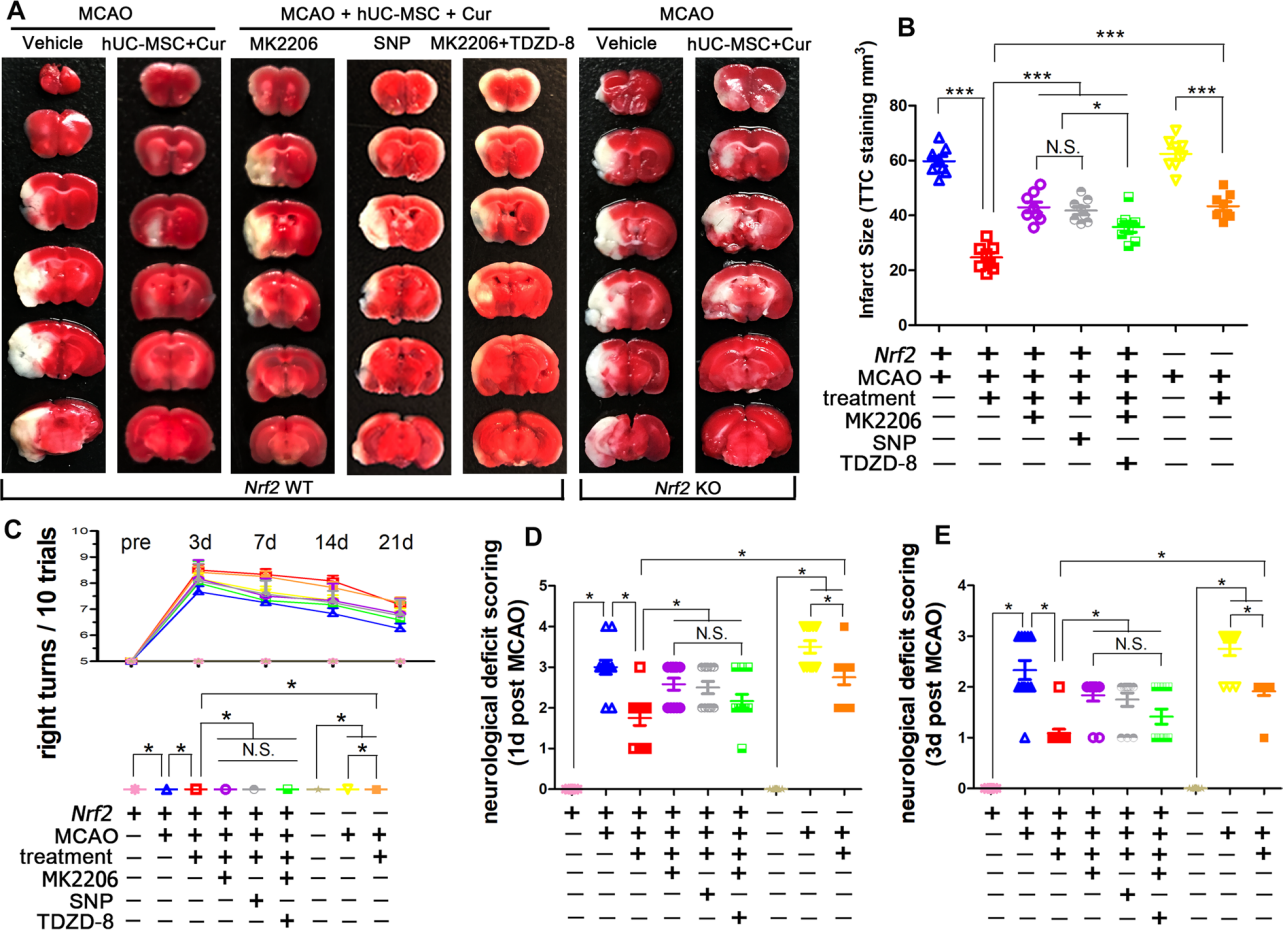
*Nrf2* knockout abolished neurological function improvement mediated by combined curcumin-hUC-MSC treatment. **A**, **B** TTC-stained brain sections (**A**) and quantitative analysis (**B**) showing the decreased infarct volume with combined therapy in MCAO mice, N = 8. **C** The corner test was performed to assess neurological deficits from pre-MCAO to 21 d post MCAO injury, N = 12. **D**, **E** The neurological deficit score (NDS) on 1 d post MCAO (**D**) and 3 d post MCAO (**D**), N = 12. *Cur* curcumin, *N.S.* no significant difference, *P < 0.05, ***P < 0.001

Finally, the therapeutic effects of the combined curcumin-hUC-MSC treatment were examined in *Nrf2*-deficient ICR mice. The TTC staining (Fig. 10A–B) showed that the combined therapy reduced the infarction volume in MCAO mice, which was consistent with previous experiments. The infarction volume in the wild type MCAO and MCAO + hUC-MSCs + curcumin groups was (in mm^3^) 59.78 ± 5.06 and 24.70 ± 4.62, respectively. The differences in the infarction volume between the combined therapy WT (24.70 ± 4.62 mm^3^) and combined therapy *Nrf2* KO (35.86 ± 5.54 mm^3^) groups demonstrated that the combined therapy reduced the infarct volume through the Nrf2 signaling pathway. Similarly, combined treatment followed with the inhibition of AKT with MK2206 or activation of GSK3β with SNP increased infarction volume, whereas further suppression of GSK3β (MK2206 + TDZD-8) reduced infarction volume. Furthermore, both right turns rate (Fig. 10C) and Neurological deficit score (Fig. 10D–E) for combined therapy WT mice decreased compared with that *Nrf2* knockout mice receiving the combined therapy. Collectively, these results substantiated that combined curcumin-hUC-MSC treatment improved neurological function by AKT/GSK-3β/β-TrCP/Nrf2 axis.

## Discussion

In this study, we found that the combined hUC-MSCs transplantation and curcumin treatment can inhibit neuron apoptosis, decrease infarct volumes and reduce motor function deficits in MCAO mice. The mechanism underlying the combined therapy involves anti-oxidative stress and anti-inflammation effects mediated by polarizing microglia into anti-inflammatory phenotype. Additionally, inhibition of AKT phosphorylation at Ser473 or promoting GSK3β phosphorylation at Ser9 or *Nrf2* knockout abolished the neuroprotective and anti-inflammatory and anti-oxidative effects mediated by combined curcumin-hUC-MSC treatment. Consequently, our data substantiated that combined curcumin-hUC-MSC treatment can promote neurological recovery through polarizing microglia into anti-inflammatory phenotype mediated the AKT/GSK-3β/β-TrCP/Nrf2 signaling pathway in AIS.

MSCs have pleiotropic mechanisms, and hence may be an ideal “multi-target” treatment option for stroke [31]. Earlier studies have reported that MSCs can differentiate into neuronal cells and form neuronal circuits with host neurons [32]. Additionally, MSC transplantation has been shown to promote neurogenesis, accelerate angiogenesis, inhibit neuronal apoptosis, and modulate neuro-immunity [13, 33, 34]. Notably, hUC-MSCs exhibit rapid proliferation and low tumorigenicity properties and act as a potent source of MSCs. However, on account of the harsh post injury microenvironment after AIS, only a very small fraction of the grafted cells may eventually exert the therapeutic effect. Curcumin is involved in various biological functions and possesses anti-inflammatory and anti- apoptosis effects. In our previous research, we have found curcumin improved hUC-MSCs survival via ERK1/2 pathway after spinal cord injury [25]. In the current study, we evaluated whether curcumin could strengthen the therapeutic efficacy of engrafted hUC-MSCs in MCAO model. We found that hUC-MSC transplantation reduced neuronal apoptosis and decreased neurological deficits, which is consistent with previous studies [9, 35]. We also discovered that combined curcumin-hUC-MSC treatment protected neurons from apoptosis, suppressed cerebral edema, diminished infarction volumes, and improved functional outcomes significantly to a greater extent at each time point than hUC-MSC transplantation alone. Taken together, these results demonstrate that curcumin could enhance the neuroprotective efficacy of hUC-MSC transplantation.

Researchers generally believe that the neuroprotective role of hUC-MSCs is mediated by paracrine mechanisms rather than their capacity to replace neural lineage cells in AIS. MSCs secrete multiple neurotrophic factors to promote endogenous neural restoration in the ischemic cerebrum [36]. In addition, intravenous administration of hUC-MSCs effectively downregulated the expression of pro-inflammatory cytokines, including IL-1β, TNF-α, matrix metalloproteinase 9, and IL-6, which are upregulated by ischemia [9]. Our data revealed that hUC-MSCs decreased the expression of IL-1β, TNF-α, and IL-6, while increasing the expression of IL-4, IL-10, and TGFβ1, verifying that hUC-MSCs have an immunoregulatory effect. Notably, in comparison to solo hUC-MSC grafting, the combined curcumin-hUC-MSC treatment significantly inhibited pro-inflammatory cytokines and promoted anti-inflammatory cytokine secretion, further demonstrating that curcumin could enhance the immune-modulating properties mediated by hUC-MSCs.

As resident immune cells in the brain, microglia polarize into classically activated M1 or alternatively activated M2 phenotype to modulate neuro-immunity after ischemia [37]. M1-like microglia secretes pro-inflammatory cytokines, such as iNOS, IL-1β, and TNF-α, and cause tissue injury. In contrast, M2-like microglia secretes anti-inflammatory cytokines, such as Arg-1, IL-4, and IL-10, sustaining neural restoration [38]. Although the M1 and M2 categories have been helpful for conceptualizing microglia activities in vitro, complex high-content experiment and multi-omics technologies, including transcriptomic, epigenomic, and proteomics, have found novel microglia polarization states beyond the standard M1/M2 dichotomy, which leads to a fierce debate in microglial M1/M2 polarization in recent years [39]. Moreover, single-cell RNA-seq studies observed the mixed phenotype at the single-cell level, not as a consequence of an admixture of polarized cells [40]. Microglia usually undergo distinctly programmed metabolic changes to exhibit diverse function and phenotype in response to various environmental and cellular stresses [41]. Improved anti-inflammatory microglia could attenuate ischemic brain injury and promoted neuronal survival [42]. Here, we found that hUC-MSC transplantation increased the expression of anti-inflammatory biomarkers Arg1 and CD206, whereas it decreased the expression of pro-inflammatory biomarkers CD86 and iNOS. These results demonstrated that hUC-MSCs promoted anti-inflammatory phenotype microglia in MCAO mice, which is consistent with previous studies [9, 43, 44]. Furthermore, the combination of hUC-MSC transplantation with cur significantly increased the percentage of anti-inflammatory phenotype microglia, further confirming that cur could reinforce the immune-modulating properties mediated by hUC-MSCs.

Next, we evaluated the main underlying mechanisms for microglial polarization mediated with combined treatment. Nrf2 is a basic leucine zipper transcription factor that not only plays a crucial role against oxidative stress, but also negatively regulates inflammatory responses, and is therefore considered a promising therapeutic target for the treatment of stroke and inflammation-associated diseases [45]. MSCs have been reported to activate Nrf2 to improve mitochondrial function, inhibit inflammation, and withstand oxidative stress response [46–48]. Although activated Nrf2 shifted M1/M2 polarization [49], the data for the effect of Nrf2 on microglial polarization mediated by hUC-MSCs was still lacking. In microglia, *Nrf2* deficiency promotes inflammatory marker expression (IL-6, Cox2, and iNOS), while inhibiting anti-inflammatory marker expression (Arg 1, IL-4) [50]. We found that the elevation of Nrf2 expression was accompanied by an increase anti-inflammatory microglia percentage after hUC-MSC transplantation, indicating that Nrf2 is involved in the microglia polarization. *Nrf2* knockout reduced the expression of anti-inflammatory microglia markers, accompanied by pro-inflammatory microglia marker synthesis inhibition. The combined treatment significantly accelerated Nrf2 expression, followed by an increase in the percentage of the anti-inflammatory phenotype, while *Nrf2* knockout reversed these phenomena. These data reveal that the combined treatment polarized microglia to the anti-inflammatory phenotype through the Nrf2 signaling pathway and that the immune modulating properties of combined treatment are mediated by paracrine mechanisms.

In addition to the classical Keap1 pathway, the exclusion and degradation of Nrf2 could by mediated by GSK3β phosphorylation in a Keap1-independent manner. GSK3β is inactivated by phosphorylation of serine, and its activity is increased by phosphorylation of tyrosine. Increasing researches reported that AKT phosphorylation at Ser473 could mediate GSK3β phosphorylation at Ser9 (indicated an inactive status) and inhibit β-TrCP ubiquitination, resulting in the export of Nrf2 from the nucleus to the cytoplasm [30, 51]. In this study, we have found that combined treatment increased the expression of AKT and GSK3β, decreased β-TrCP nuclear accumulation and enhanced Nrf2 activation. These data indicate that AKT/GSK-3β/β-TrCP axis may be involved in the Nrf2-mediated antioxidant and anti-neuroinflammatory activities upon combined treatment. Therefore, we used the AKT inhibitor MK2206, the GSK3β activator SNP and inhibitor TDZD-8 to explore the relationship between AKT/GSK-3β/β-TrCP axis and Nrf2 in combined treatment. After AKT inhibition with MK2206, the activity of GSK-3β increased and the promoted the β-TrCP nuclear translocation, which resulted in the suppression of Nrf2 activity. In contrast, further reduced GSK-3β phosphorylation (activation) after ATK inhibition by TDZD-8 could suppress the β-TrCP nuclear accumulation and thereby enhance the expression of Nrf2. Moreover, upregulated GSK-3β phosphorylation (inactivation) after AKT activation could reverse the above phenomenon. All of these results demonstrate that the combined treatment promote Nrf2 expression via Akt/GSK3β/β-TrCP axis.

Intriguingly, we found that combined therapy significantly enhanced the neurological function improvement by polarizing anti-inflammatory phenotype microglia to a greater extent than the solo hUC-MSC transplantation. However, *Nrf2* knockout did not abolish the neuroprotective effect absolutely, suggesting that there may be other signaling pathways enlisted by combined therapy for promoting anti-inflammatory microglia polarization, such as endogenous interleukin-1 receptor antagonist [9]. Overall, hUC-MSC transplantation promoted recovery by the Akt/GSK3β/β-TrCP/Nrf2 signaling pathway, and curcumin enhanced the therapeutic efficacy of engrafted hUC-MSCs in experimental AIS.

In this manuscript, we demonstrate the direct evidences for the hUC-MSCs cross the blood brain barrier to stroke foci due to a few cells being detected in the brain. Based on the above experiments combined with related literature, we speculate that curcumin promoted the anti-inflammatory and antioxidant effect of hUC-MSCs through the following path: (1) protecting hUC-MSCs from apoptosis, (2) enhancing hUC-MSCs survival via improved inflammatory microenvironment after stroke, (3) promoting the immunomodulatory capacity of the hUC-MSCs. The harsh inflammatory microenvironment after AIS restricts the efficacy of single treatment. This study confirmed that combination therapy can improve the prognosis of AIS through multiple approaches, providing an important therapy strategy for AIS. In the future, identified paracrine extracellular vesicle which play a neuroprotective role for hUC-MSCs is a work in progress.

## Conclusions

In summary, the present study has demonstrated that curcumin can reinforce the therapeutic efficacy of engrafted hUC-MSCs in experimental MCAO mice. The combined curcumin-hUC-MSC treatment effectively protected neurons from apoptosis, reduced infarction volumes, and improved the outcomes of ischemic stroke. The neuroprotective mechanism of this combined therapy was mediated by the polarized microglia of anti-inflammatory phenotype via AKT/GSK-3β/β-TrCP/Nrf2 axis. Thus, we have performed a comprehensive preclinical experiment for combined curcumin-hUC-MSC therapy in mouse model. This therapy can potentially be an effective treatment for AIS in humans.

## Supplementary Information

**Additional file 1**: **Figure S1:** Cytotoxicity of curcumin on microglia and hUC-MSC. To determine the optimal concentration of curcumin, we assessed the cytotoxicity of curcumin on microglia and hUC-MSCs after treatmentwith different concentrations of curcumin for 24 h by the CCK-8. For the microglia,there were significant differences in viability when the concentration ofcurcumin was ≥ 16 μmol/L compared with that of the control group, indicating nocytotoxicity when the concentration of curcumin was ≤ 8 μmol/L. For the hUC-MSC, there was no cytotoxicity when the curcumin concentration was ≤ 4μmol/L. Therefore, we selected 4 μmol/L as the optimum concentration ofcurcumin for subsequent experiments. N = 5; N.S., no significant difference, *P < 0.05. **Figure S2:** Characterization ofhUC-MSCs. The flow cytometry revealed that the phenotypes of mesenchymal stemcells were positive for CD73, CD90 and CD105, while the phenotypes ofhematopoietic stem cells were negative for CD34, CD45 and HLA-DR. **Figure S3: **Tracing the migration of the hUC-MSC in the CNS. (A) Representative images of immunofluorescence analysis for hUC-MSC identification, hUC-MSC labeled with MAB1281, STRO-1 and CD44, respectively. (B-D) Quantitative analysisof MAB1281^+^ cells (B), STRO-1^+^ cells (C) and CD44^+^cells (D) in the ipsilateral peri-infarct. Cur: curcumin, scale bars = 50 μm,N=3, ***P < 0.001. **Figure S4: **Combined curcumin-hUC-MSC treatment promoted anti-inflammatory phenotype microglia in MCAO mice. (A-B) Representative images of immunofluorescence analysis formicroglia polarization, (A) The microglial cell-specific marker Iba1 (green), pro-inflammatorymicroglia marker CD86 (red), nuclei (blue); (B) the microglial cell-specificmarker Iba1 (green), anti-inflammatory microglia marker Arg1 (red), nuclei(blue); (C-D) Quantitative analysis of pro-inflammatory (C) and anti-inflammatory(D) microglia in the ipsilateral peri-infarct. Cur: curcumin, scale bars = 20μm; N = 5; N.S., no significant difference, *P < 0.05, **P < 0.01, ***P< 0.001. **Figure S5: ***Nrf2* involvement in the anti-inflammatoryphenotype microglia polarization mediated by combined curcumin-hUC-MSC treatmentin MCAO mice. (A-C) Representative flow cytometric (A) and quantitative datafor the percentage of CD86^+^ cells (B) and CD206^+^ (C) microglia in the ipsilateral peri-infarct isolated from MCAO mice subjected todifferent treatments. (D-G) qRT-PCR analysis of pro-inflammatory microglia gene *iNOS* (D) and* CD86* (E) and anti-inflammatory microglia gene *Arg1* (F) and *CD206* (G). Combined curcumin-hUC-MSC treatment decreased the pro-inflammatory marker expressionand increased anti-inflammatory marker expression. Inhibition of AKT with MK2206 or activation of GSK3β with SNP increased pro-inflammatory subtype marker expression and decreased the anti-inflammatory marker expression, whereas further suppression of GSK3β (MK2206+TDZD-8) enhanced anti-inflammatory marker expression at both protein and mRNA levels. Furthermore, *Nrf2* knockout abolished the anti-inflammatorymicroglia phenotypic polarization mediated by the combined treatment. Cur,curcumin, N = 5; N.S., no significant difference, **P < 0.01, ***P <0.001.

## Data Availability

The datasets used and/or analyzed during the current study are available from the corresponding author on reasonable request.

## Abbreviations

AIS: Acute ischemic stroke
AKT: Protein kinase B
ARE: Antioxidant response elements
Arg1: Arginase-1
BBB: Blood brain barrier
BCA: Bicinchoninic acid protein
β-TrCP: Beta-transducing repeat-containing protein
CBF: Cerebral blood flow
CCK-8: Cell counting kit-8
CC3: C-caspase-3
CM: Conditioned medium
CNS: Central nervous system
ELISA: Enzyme-linked immunosorbent assay
FACS: Fluorescence-activated cell sorting
DMSO: Dimethyl sulfoxide
ELISA: Enzyme-linked immunosorbent assay
FACS: Fluorescence-activated cell sorting
GSK-3β: Glycogen synthase kinase 3 beta
HO-1: Hemeoxygenase
hUC-MSC: Human umbilical cord-derived mesenchymal stem cell
ICR: Institute of Cancer Research
IL-1β: Interleukin-1 beta
IL-4: Interleukin-4
IL-6: Interleukin-6
IL-10: Interleukin-10
iNOS: Inducible nitric oxide synthase
Keap1: Kelch-like ECH-associated protein 1
KO: Knockout
MCAO: Middle cerebral artery occlusion
MK2206: 8-[4-(1-Aminocyclobutyl)phenyl]-9-phenyl-2H-[1,2,4]triazolo[3,4-f][1,6]naphthyridin-3-one
MMP9: Matrix metalloproteinase 9
MSCs: Mesenchymal stem cells
BM: Mesenchymal stem cell basal medium
NDS: Neurological deficit score
NQO1: NAD(P)H dehydrogenase (quinone 1)
Nrf2: Transcription factor nuclear factor-erythroid 2-related factor 2
OD: Optical density
OGD: Oxygen glucose deprivation
PVDF: Polyvinylidene fluoride
RT-PCR: Real-time polymerase chain reaction
SD: Standard deviation
SDS-PAGE: Sodium dodecyl sulfate polyacrylamide gel electrophoresis
SNP: Sodium nitroprusside
TDZD-8: 4-Benzyl-2-methyl-1,2,4-thiadiazolidine-3,5-dione
TGF-β1: Transforming growth factor-β1
TNF-α: Tumor necrosis factor alpha
TTC: 2,3,5-Triphenyl tetrazolium chloride
WT: Wild-type
