# Supplementary material for: Human umbilical cord-derived mesenchymal stem cell transplantation supplemented with curcumin improves the outcomes of ischemic stroke via AKT/GSK-3β/β-TrCP/Nrf2 axis
Source: J Neuroinflammation. 2023 Feb 24;20:49. doi: 10.1186/s12974-023-02738-5 (PMC9951499; doi:10.1186/s12974-023-02738-5)

Human umbilical cord-derived mesenchymal stem cell transplantation supplemented with curcumin improves the outcomes of ischemic stroke via AKT/GSK-3β/β-TrCP/Nrf2 axis

**Yuan Li ^1, #^, Jialu Huang ^1, #^, Jie Wang ^1^, Simin Xia^1^, Hong Ran ^1^, Lenyu Gao ^2,1^, Chengjian Feng ^3^, Li Gui ^1^, Zhenhua Zhou ^1 *^, Jichao Yuan ^1^ ^*^**

1 Department of Neurology, Southwest Hospital, Third Military Medical University (Army Medical University), Chongqing 400038, China

2 Department of Traditional Chinese Medicine and Rheumatology, Southwest Hospital, Third Military Medical University (Army Medical University), Chongqing 400038, China

3 Department of Medical Engineering，958th Hospital of the People's Liberation Army, Chongqing 400038, China

# These authors contributed equally to this work.

*** Correspondence:**

**Corresponding author Yuan Jichao,** [**yuanjichao_tmmu**](mailto:yuanjichao@tmmu.edu.cn)**@163.com, 29 Gaotanyan Street, Chongqing 400038, China, Tel.: +86 21 68765471**

**Corresponding author Zhou Zhenhua,** [**exploiter001@126.com**](mailto:exploiter001@126.com)**，29 Gaotanyan Street, Chongqing 400038, China, Tel.: +86 21 68765461**

**Figure S1:** Cytotoxicity of curcumin on microglia and hUC-MSC. To determine the optimal concentration of curcumin, we assessed the cytotoxicity of curcumin on microglia and hUC-MSCs after treatment with different concentrations of curcumin for 24 h by the CCK-8. For the microglia, there were significant differences in viability when the concentration of curcumin was ≥ 16 μmol/L compared with that of the control group, indicating no cytotoxicity when the concentration of curcumin was ≤ 8 μmol/L. For the hUC-MSC, there was no cytotoxicity when the curcumin concentration was ≤ 4 μmol/L. Therefore, we selected 4 μmol/L as the optimum concentration of curcumin for subsequent experiments. N = 5; N.S., no significant difference, *P < 0.05.


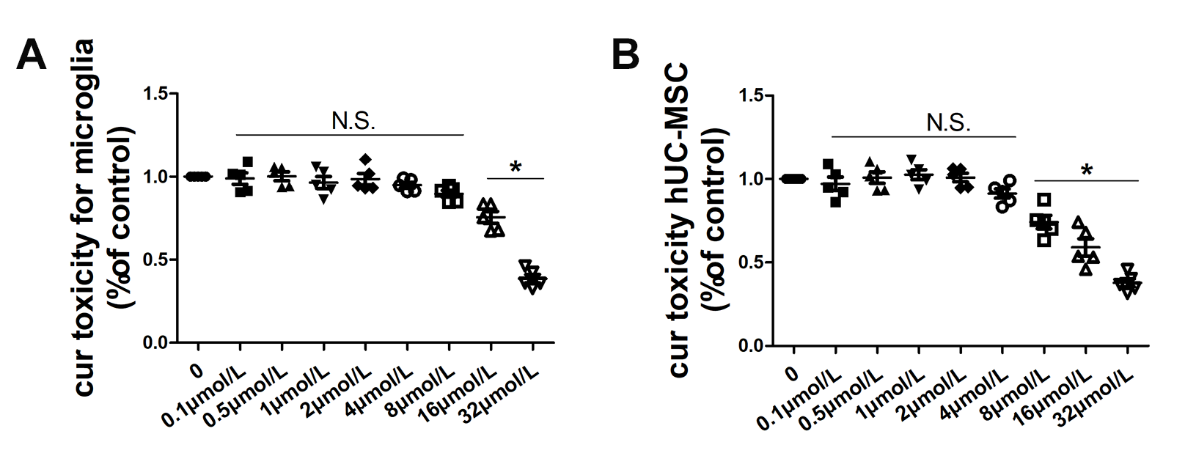


**Figure S2:** Characterization of hUC-MSCs. The flow cytometry revealed that the phenotypes of mesenchymal stem cells were positive for CD73, CD90 and CD105, while the phenotypes of hematopoietic stem cells were negative for CD34, CD45 and HLA-DR.


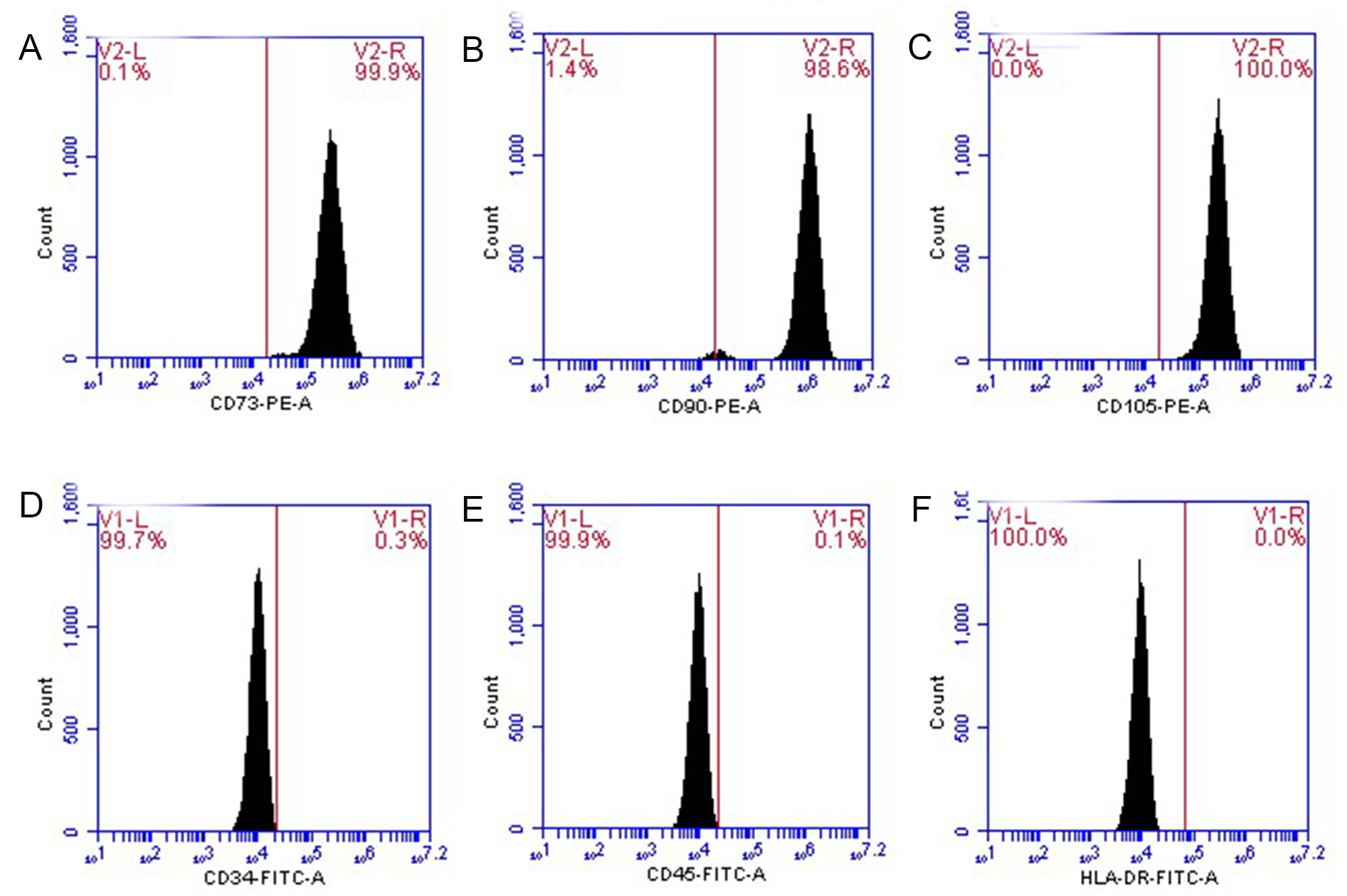


**Figure S3:** Tracing the migration of the hUC-MSC in the CNS. (A) Representative images of [immunofluorescence](javascript:;) analysis for hUC-MSC identification, hUC-MSC labeled with MAB1281, STRO-1 and CD44, respectively. (B-D) Quantitative analysis of MAB1281^+^ cells (B), STRO-1^+^ cells (C) and CD44^+^ cells (D) in the ipsilateral peri-infarct. Cur: curcumin, scale bars = 50 μm, N=3, ***P < 0.001.


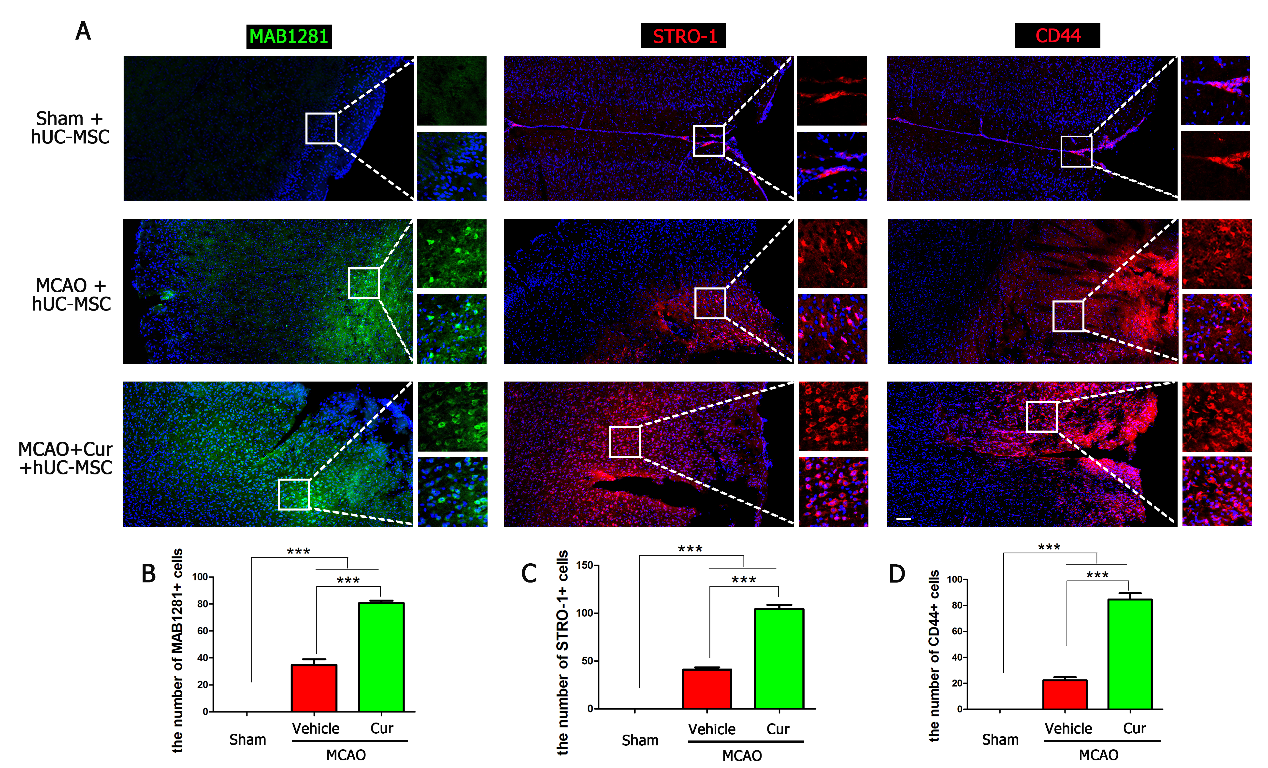


**Figure S4:** Combined curcumin-hUC-MSC treatment promoted anti-inflammatory phenotype microglia in MCAO mice. (A-B) Representative images of [immunofluorescence](javascript:;) analysis for microglia polarization, (A) The microglial cell-specific marker Iba1 (green), pro-inflammatory microglia marker CD86 (red), nuclei (blue); (B) the microglial cell-specific marker Iba1 (green), anti-inflammatory microglia marker Arg1 (red), nuclei (blue); (C-D) Quantitative analysis of pro-inflammatory (C) and anti-inflammatory (D) microglia in the ipsilateral peri-infarct. Cur: curcumin, scale bars = 20 μm; N = 5; N.S., no significant difference, *P < 0.05, **P < 0.01, ***P < 0.001.


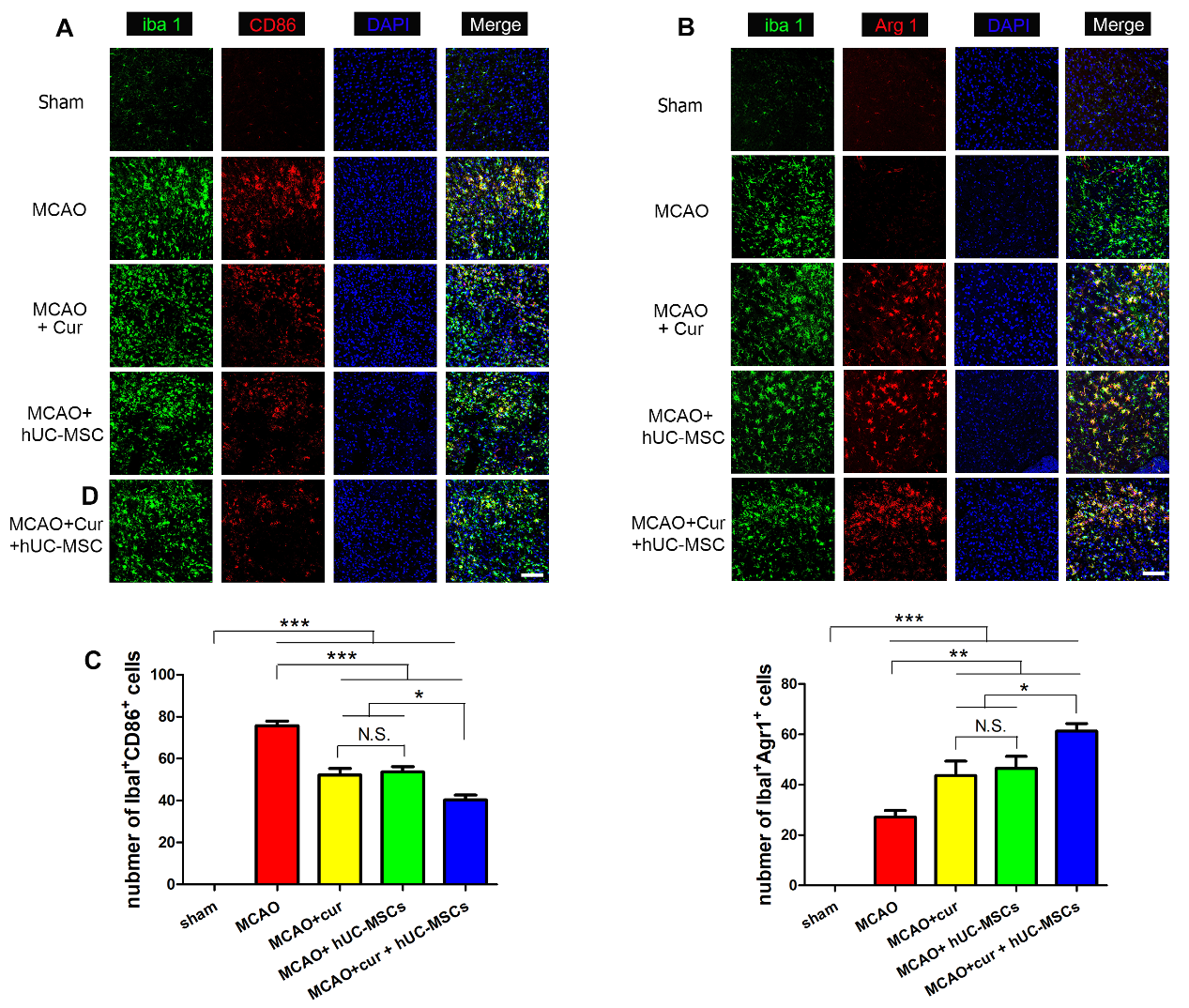


**Figure S5:** *Nrf2* involvement in the anti-inflammatory phenotype microglia polarization mediated by combined curcumin-hUC-MSC treatment in MCAO mice. (A-C) Representative flow cytometric (A) and quantitative data for the percentage of CD86^+^ cells (B) and CD206^+^ (C) microglia in the ipsilateral peri-infarct isolated from MCAO mice subjected to different treatments. (D-G) qRT-PCR analysis of pro-inflammatory microglia gene *iNOS* (D) and *CD86* (E) and anti-inflammatory microglia gene *Arg1* (F) and *CD206* (G). Combined curcumin-hUC-MSC treatment decreased the pro-inflammatory marker expression and increased anti-inflammatory marker expression. Inhibition of AKT with MK2206 or activation of GSK3β with SNP increased pro-inflammatory subtype marker expression and decreased the anti-inflammatory marker expression, whereas further suppression of GSK3β (MK2206+TDZD-8) enhanced anti-inflammatory marker expression at both protein and mRNA levels. Furthermore, *Nrf2* knockout abolished the anti-inflammatory microglia phenotypic polarization mediated by the combined treatment. Cur, curcumin, N = 5; N.S., no significant difference, **P < 0.01, ***P < 0.001.


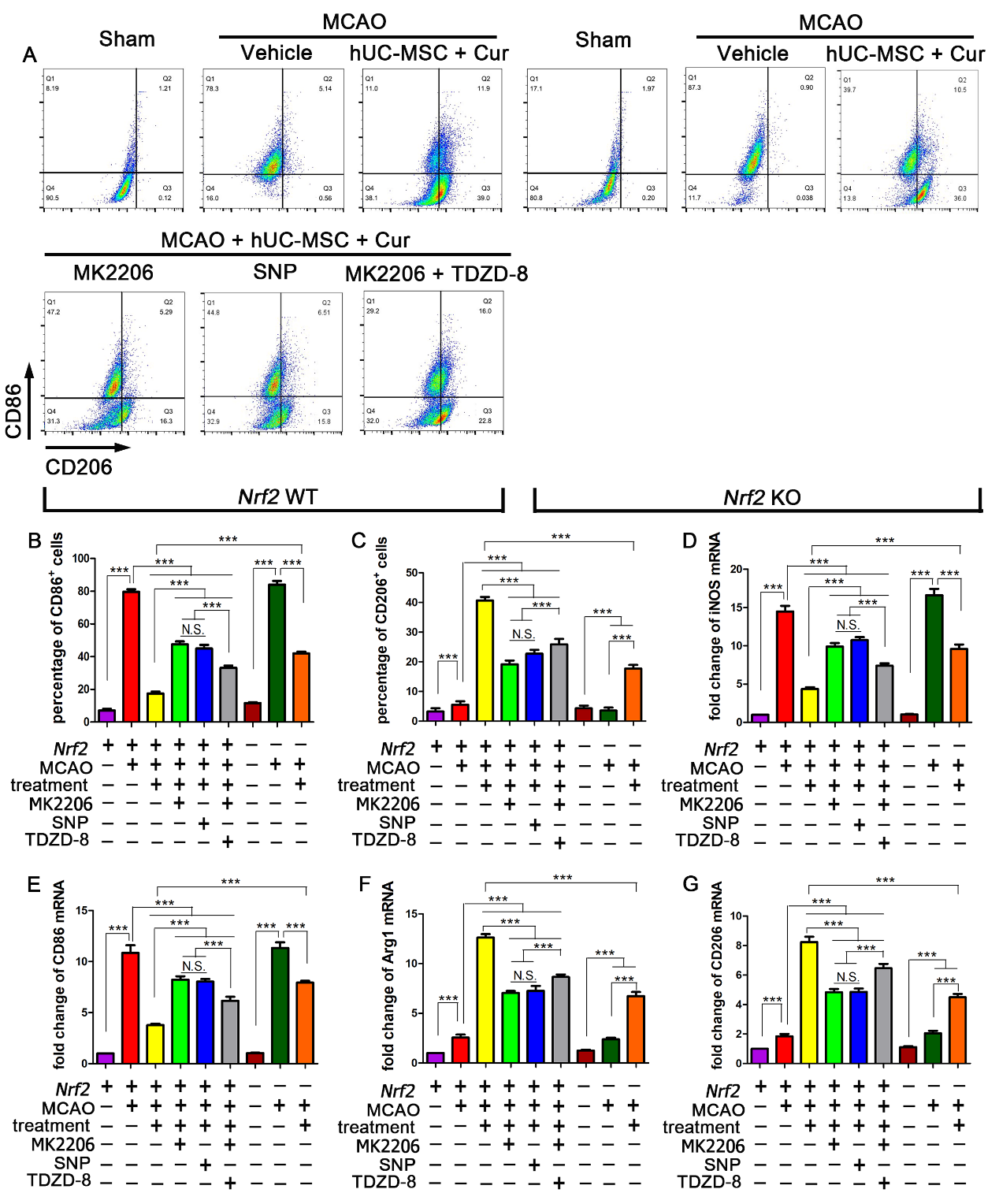

Supplement: Supplementary file 1 — Additional file 1: Figure S1: Cytotoxicity of curcumin on microglia and hUC-MSC. To determine the optimal concentration of curcumin, we assessed the cytotoxicity of curcumin on microglia and hUC-MSCs after treatmentwith different concentrations of curcumin for 24 h by the CCK-8. For the microglia,there were significant differences in viability when the concentration ofcurcumin was ≥ 16 μmol/L compared with that of the control group, indicating nocytotoxicity when the concentration of curcumin was ≤ 8 μmol/L. For the hUC-MSC, there was no cytotoxicity when the curcumin concentration was ≤ 4μmol/L. Therefore, we selected 4 μmol/L as the optimum concentration ofcurcumin for subsequent experiments. N = 5; N.S., no significant difference, *P < 0.05. Figure S2: Characterization ofhUC-MSCs. The flow cytometry revealed that the phenotypes of mesenchymal stemcells were positive for CD73, CD90 and CD105, while the phenotypes ofhematopoietic stem cells were negative for CD34, CD45 and HLA-DR. Figure S3: Tracing the migration of the hUC-MSC in the CNS. (A) Representative images of immunofluorescence analysis for hUC-MSC identification, hUC-MSC labeled with MAB1281, STRO-1 and CD44, respectively. (B-D) Quantitative analysisof MAB1281+ cells (B), STRO-1+ cells (C) and CD44+cells (D) in the ipsilateral peri-infarct. Cur: curcumin, scale bars = 50 μm,N=3, ***P < 0.001. Figure S4: Combined curcumin-hUC-MSC treatment promoted anti-inflammatory phenotype microglia in MCAO mice. (A-B) Representative images of immunofluorescence analysis formicroglia polarization, (A) The microglial cell-specific marker Iba1 (green), pro-inflammatorymicroglia marker CD86 (red), nuclei (blue); (B) the microglial cell-specificmarker Iba1 (green), anti-inflammatory microglia marker Arg1 (red), nuclei(blue); (C-D) Quantitative analysis of pro-inflammatory (C) and anti-inflammatory(D) microglia in the ipsilateral peri-infarct. Cur: curcumin, scale bars = 20μm; N = 5; N.S., no significant difference, *P < 0.0 [file 12974_2023_2738_MOESM1_ESM.docx]
